# In-Depth Characterization of *bZIP* Genes in the Context of Endoplasmic Reticulum (ER) Stress in *Brassica campestris* ssp. *chinensis*

**DOI:** 10.3390/plants13081160

**Published:** 2024-04-22

**Authors:** Aliya Ayaz, Abdul Jalal, Xiaoli Zhang, Khalid Ali Khan, Chunmei Hu, Ying Li, Xilin Hou

**Affiliations:** 1 State Key Laboratory of Crop Genetics and Germplasm Enhancement, Ministry of Science and Technology/National Key Laboratory of Crop Genetics and Germplasm Enhancement, Key Laboratory of Horticultural Crop Biology and Genetic Improvement (East China) of MOA, College of Horticulture, Nanjing Agricultural University, Nanjing 210095, China; 2 Biofuels Institute, School of Emergency Management, School of the Environment and Safety Engineering, Jiangsu University, Zhenjiang 212013, China; 3 Applied College, Center of Bee Research and Its Products (CBRP), Unit of Bee Research and Honey Production, and Research Center for Advanced Materials Science (RCAMS), King Khalid University, Abha 61413, Saudi Arabia

**Keywords:** *BcbZIP* genes, phylogeny, gene interaction network, expression profiles, functional analysis, ER stress

## Abstract

Numerous studies have been conducted to investigate the genomic characterization of *bZIP* genes and their involvement in the cellular response to endoplasmic reticulum (ER) stress. These studies have provided valuable insights into the coordinated cellular response to ER stress, which is mediated by *bZIP* transcription factors (TFs). However, a comprehensive and systematic investigations regarding the role of *bZIP* genes and their involvement in ER stress response in pak choi is currently lacking in the existing literature. To address this knowledge gap, the current study was initiated to elucidate the genomic characteristics of *bZIP* genes, gain insight into their expression patterns during ER stress in pak choi, and investigate the protein-to-protein interaction of *bZIP* genes with the ER chaperone BiP. In total, 112 members of the *BcbZIP* genes were identified through a comprehensive genome-wide analysis. Based on an analysis of sequence similarity, gene structure, conserved domains, and responsive motifs, the identified *BcbZIP* genes were categorized into 10 distinct subfamilies through phylogenetic analysis. Chromosomal location and duplication events provided insight into their genomic context and evolutionary history. Divergence analysis estimated their evolutionary history with a predicted divergence time ranging from 0.73 to 80.71 million years ago (MYA). Promoter regions of the *BcbZIP* genes were discovered to exhibit a wide variety of *cis*-elements, including light, hormone, and stress-responsive elements. GO enrichment analysis further confirmed their roles in the ER unfolded protein response (UPR), while co-expression network analysis showed a strong relationship of *BcbZIP* genes with ER-stress-responsive genes. Moreover, gene expression profiles and protein–protein interaction with ER chaperone *BiP* further confirmed their roles and capacity to respond to ER stress in pak choi.

## 1. Introduction

Transcription factors (TFs) are the main players in regulating gene expression, serving essential functions in many biological processes within plants [1,2]. Until now, many TF families have been reported in plants [3]. One of the largest TFs family is the basic leucine zipper (*bZIP*) TF family [4,5]. Plant *bZIP* TFs family (*bZIP* genes) have diverse roles in developmental and physiological processes. These roles include ABA signaling for osmotic stress responses during vegetative growth [6], flowering time and seed germination [7], glucose-ABA signaling [8] lipid stress responses [9], response to zinc deficiency [10], sugar signaling during metabolism [11], activation of SA-dependent plant systemic defense responses, and JA- and ethylene-dependent defense mechanisms [12]. Furthermore, *bZIP* genes have been implicated in the modulation of signal transduction pathways and responses to a wide range of stresses. These stresses encompass drought, high salinity, cold stress, pathogen infection, osmotic stress, as well as ER stress [13,14,15]. Endoplasmic reticulum (ER) stress occurs when cells encounter an inequilibrium between the capacity to fold proteins and the demand for protein folding, resulting in the buildup of unfolded or misfolded proteins within the ER [16]. The *bZIP* genes play a vital role in regulating the UPR during ER stress [17]. The UPR is a cellular mechanism that is triggered when there is an accumulation of improperly folded or unfolded proteins within the ER [18].

In plants, *bZIP* genes have emerged as key regulators in response to ER stress, ensuring cellular homeostasis and survival under these challenging conditions [19]. The UPR in plants encompasses two functionally overlapping branches: *IRE1*-*bZIP60* and *bZIP17*/*bZIP28* [20], which are strongly associated with ER-responsive genes. The first branch of UPR involved *IRE1*, an ER receptor [21], acts as an endoribonuclease and a serine/threonine-protein kinase [22]. It mediates two distinct signaling pathways to regulate translation overload: the unconventional splicing of the *bZIP60*, and RNA cleavage through RIDD [23,24]. The N-terminal of *IRE1* acts as a sensor for misfolded proteins, while the C-terminal serves as an RNA processing enzyme, specifically involved in unconventional *bZIP60* splicing, a crucial regulator of the ER stress response [25]. While in the second branch, the *bZIP17*/*bZIP28* proteins are characterized as type II transmembrane proteins, featuring a cytosolic N-terminal region containing the *bZIP* TF domain, while their C-terminus in the ER lumen ensures their retention within the ER. Under normal conditions, *BiP* interacts with *bZIP17*/*28*, retaining them in the ER [26]. However, during ER stress, *BiP* dissociates, facilitating the translocation of *bZIP17*/*28* from ER to Golgi complex [27]. Subsequently, *bZIP28* is cleaved by an unidentified protease, enabling the functioning of S2P. Conversely, S1P cleaves *bZIP17*. The cleaved forms of *bZIP17*/*bZIP28* then translocated to the nucleus and form transcriptional complexes with nuclear TFs, leading to the activation of UPR genes [28]. Notably, both *bZIP17*/*bZIP28* exhibit comparable characteristics and respond to various stimuli, including ER stress inducers, environmental cues, and developmental factors. These findings shed light on the intricate molecular mechanisms underlying the UPR in plants, emphasizing the interplay between *bZIP* genes and ER-stress-responsive genes.

BiP, on the other hand, is a binding protein abundant in the ER lumen, possesses an ATP-binding domain at its N-terminus, and a protein-binding domain at its C-terminus. This C-terminal domain enables BiP to bind to the hydrophobic surfaces of nascent proteins, thereby protecting them from aggregation through an ATP-dependent mechanism [29,30]. In response to ER stress induced by adverse environmental factors like heat and drought, or specific ER stressors such as TM and DTT, the expression of *BiP* genes is up-regulated through the UPR [22]. The overexpression of *BiP* genes has been demonstrated to enhance plant tolerance to environmental stresses. For instance, the overexpression of *BiP* genes is discovered along with the application of exogenous chemical chaperones such as sodium 4-phenylbutyrate (PBA), mitigated ER stress induced by DTT and high temperatures [31]. Moreover, the overexpression of soybean *BiP* in tobacco plants conferred tolerance to water deficit during plant growth by preventing endogenous oxidative stress [32]. The silencing of *BiP* genes in tomato compromised Ve1-mediated resistance to *Verticillium dahlia* [33], and the overexpression of *BiP* in soybean and tobacco plants resulted in hypersensitivity to *Pseudomonas syringae* pv tomato [34].

This study aimed to explore the genomic characteristics, expression patterns, and functional implications of *BcbZIP* genes in the context of ER stress response in pak choi (*Brassica campestris* ssp. *chinensis*), a leafy vegetable belonging to the *Brassicaceae* family. Pak choi is widely cultivated and consumed due to its nutritional value and culinary properties [35,36]. Understanding the molecular mechanisms underlying the ER stress response in pak choi can provide valuable insights into enhancing its stress tolerance and improving its overall quality. The objectives of this study are threefold. Firstly, we aimed to characterize the genomic organization and structural features of *BcbZIP* gene family in pak choi. This involved a genome-wide identification, physicochemical properties, phylogenetic analysis, gene structure organization, conserved domains and motif analysis, chromosomal arrangement and duplication events, divergence analysis, *cis*-element analysis, and GO enrichment analysis. Further protein–protein interaction network of *BcbZIP* genes with ER stress-responsive genes was also performed via string interaction network analysis. Secondly, we investigated the expression profiles of the *BcbZIP* genes in response to ER stress using RNA seq data (unpublished). Lastly, we experimentally investigated the role of *BcbZIP* genes in ER stress response. This involved subcellular localization and protein–protein interaction, both *in vitro* and *in vivo*, to explore the interaction and binding sites of *BcbZIP60a* and *BcbZIP60b* with ER chaperone *BcBiP3*, providing insights into the regulatory networks and signaling pathways involved in ER stress response in Pak choi.

## 2. Results

### 2.1. Total 112 BcbZIP Genes Were Identified and Their Physicochemical Properties Were Evaluated

In this study, 112 *bZIP* in pak choi termed as *BcbZIP*, after removing redundant sequences, were identified using HMM and BLASTp searches (see Materials and Methods). In order to comprehensively investigate the physicochemical characteristics of 112 identified *BcbZIP* genes, various parameters were evaluated *in silico*, and the results are presented in Supplementary File S1. The parameters analyzed included gene ID, location on chromosome, amino acids and CDS length, molecular weight (MW/kDa), isoelectric point (PI), GRAVY, formula, and predicted subcellular location. Analysis showed that *BcbZIP* genes were distributed within the genome from chromosome 1 to chromosome 10, and their start and end positions were specifically identified. However, the start and end position of the gene *BraC06g046020.1* was not predicted. Among the *BcbZIP* genes, 57 genes were located on the reverse strand, 54 genes were located on the forward strand, while 1 gene did not have any available information in the NHCC database. Furthermore, the analysis revealed that amino acid (AA) length of *BcbZIP* genes varied from 120–780, while the CDS length ranged from 363–5728. The molecular weight (MW/kDa) of *BcbZIP* genes varied from 14,032.72–65,103.97, and the isoelectric point (PI) ranged from 4.98–10.11 PI. Additionally, the predicted subcellular localization analysis indicated that 63 *BcbZIP* genes were found in the nucleus, 33 in both the endoplasmic reticulum and nucleus, 9 in cytoplasm, 3 in the endoplasmic reticulum, and the remaining 3 genes were found in chloroplast, ER/cytoplasm, and ER/cytoplasm/nucleus, respectively. Overall, these results provide important understandings into the known basic parameters of the *BcbZIP* genes.

### 2.2. Phylogenetic Analysis Divided BcbZIP Genes into 10 Sub Families

**Figure 1:**
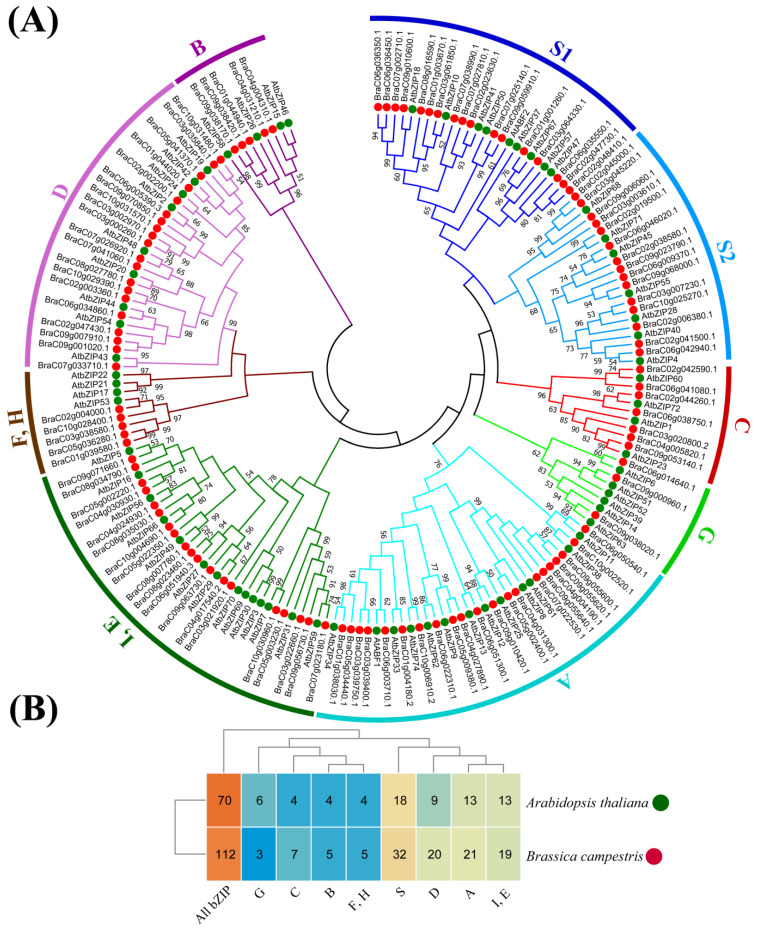
Phylogenetic analysis of *bZIP* genes in pak choi and Arabidopsis. (**A**) The phylogenetic tree divided into 10 sub families based on Arabidopsis sequences. (**B**) The heatmap of number of *bZIP* genes in each subfamily of pak choi and Arabidopsis.

In order to conduct a more comprehensive analysis of the evolutionary relationship among *BcbZIP* genes, an un-rooted phylogenetic tree was constructed using the 70 protein sequences of *AtbZIP* and 112 identified *BcbZIP* genes with 1000 bootstrap values using the neighbor joining method. The phylogenetic tree divided the *bZIP* genes into 10 sub families. i.e., A, B, C, D, E, I, S (S1 and S2), G, F, and H based on Arabidopsis sub groups. Within subfamily A, the 13 *AtbZIP* genes were clustered with the 21 *BcbZIP* genes. In subfamily B, four *AtbZIP* genes were grouped with the five *BcbZIP* genes. In subfamily C, four *AtbZIP* genes were grouped with the seven *BcbZIP* genes. In subfamily D, 9 *AtbZIP* genes were grouped with the 20 *BcbZIP* genes. In subfamilies E and I, 13 *AtbZIP* genes were grouped with the 19 *BcbZIP* genes. In subfamily S, 18 *AtbZIP* genes were grouped with the 32 *BcbZIP* genes. In subfamily G, six *AtbZIP* were grouped with the three *BcbZIP* genes, and in subfamilies F and H, four *AtbZIP* genes were grouped with the five *BcbZIP* genes. Overall, these findings offer valuable insights into the evolutionary connections between *AtbZIP* and *BcbZIP* genes (Figure 1).

### 2.3. The Chromosomal Location and Duplication Events of BcbZIP Genes

**Figure 2:**
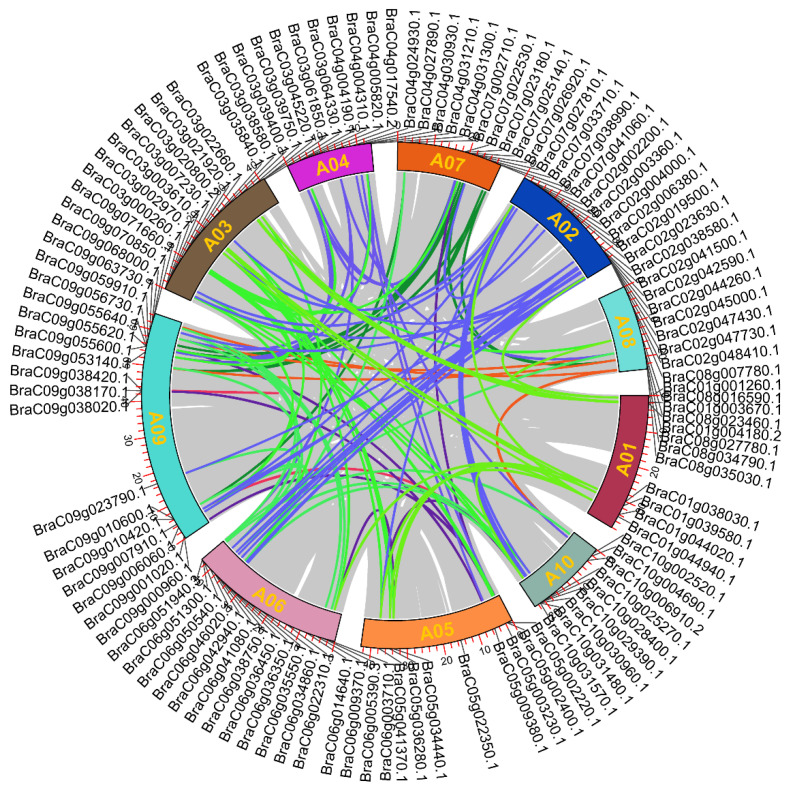
The collinearity and distribution of 112 *BcbZIP* genes. The chromosomal distribution of 112 *BcbZIP* genes are illustrated on 10 chromosomes along the circumference of the circle. The different colored lines within the circle represent collinearity relationships among *BcbZIP* genes.

Chromosomal distribution and duplication of *BcbZIP* genes were investigated and visualized on a circos plot (Figure 2). Results revealed that out of 112 *BcbZIP* genes, there was uneven distribution across the 10 chromosomes: with chromosome 1 containing 7 *BcbZIP* genes, each chromosome 2, 3, and 6 containing 14 *BcbZIP* genes, each chromosome 4, 7, and 10 containing 9 *BcbZIP* genes, chromosome 5 containing 8 *BcbZIP* genes, chromosome 8 containing 6 *BcbZIP* genes, and chromosome 9 containing 20 *BcbZIP* genes. The observed multigene families could have arisen from region-specific duplication or genome-wide polyploidization. This evolutionary mechanism has been demonstrated as a significant aspect of plant genome evolution. Additionally, 38 pairs of similar paralogues were identified and grouped in the same sub clade, implying that these gene duplicates originated from duplication events. This approach provides a reliable and standardized approach for assessing gene duplication events and their chromosomal distribution.

### 2.4. Divergence Analysis Investigated How BcbZIP Genes Have Evolved and Diversified over Time

In order to gain a better understanding of the evolutionary characteristics of *BcbZIP* genes, a divergence analysis was conducted. This involved calculating the ratio of Ka/Ks for each pair of paralogous *BcbZIP* genes. Total 38 *BcbZIP* paralogous genes were identified with a *Ka/Ks* calculator (Supplementary File S2, Figure S6). The results indicated that *Ka* divergence values for different paralogous pairs ranges from 0.001699–0.308732, *Ks* divergence values ranges from 0.017292–1.058908, while *Ka/Ks* divergence value ranges from 0.06311–1.004532. Notably, the *Ka/Ks* ratio can be utilized for assessing selective pressure on genes in molecular biology. Overall, most of the gene pairs (99.2%) had a *Ka/Ks* ratio of less than 1, showing purifying selection during the evolution of most *bZIP* genes in this family. For each paralogous pair of *BcbZIP* genes, the divergence time was found to be between 0.73–80.71 MYA (Table 1). This approach provides a valuable and standardized methodology for exploring the evolutionary dynamics of gene families and their diversification through time.

### 2.5. Gene Structure, Domains, and Motifs Analysis Provide a Genetic Basis of Biological Processes to Infer the Potential Functions of BcbZIP Genes

To better understand the basic functions of identified *BcbZIP* genes, the protein and nucleotide sequences were analyzed for conserved domains, gene structure organization, and motifs using TBtools. Pfam protein analysis and sequence analysis showed that all genes contained a *bZIP* domain. The specific *bZIP* domains found in all *BcbZIP* genes included bZIP_plant_BZIP46, bZIP superfamily, bZIP_HBP1b-like, bZIP_HY5-like, bZIP_plant_RF2, bZIP_plant_GBF1, bZIP_C superfamily, and bZIP_C. Additionally, ten different motifs were identified by using MEME suite software 5.4.1 corresponding to bZIP domains to explore their structural and functional diversity (Supplementary File S2, Figure S1). Moreover, the subfamily S (S1 and S21) contain motif 1 and 6 following three domains (bZIP plant GBF1, PRK 13,875 super family, and bZIP super family) (Supplementary File S2, Figure S2). The subfamily C, G, and A contain motif 1, 6, 7, 9, and 10 following 12 domains (bZIP C, bZiP superfamily, bZiP plant GBF1, bZIP C superfamily, AMN1 superfamily, F-box SF superfamily, MFMR, bZIP1, DUF6515 superfamily, MFMR assoc superfamily, BRLZ, and bZIP plant BZIP46) (Supplementary File S2, Figure S3). The subfamily I, E, F, H, D, and B contain motif 1, 2, 3, 4, 5, 6, 8, and 10 following 12 domains (bZiP plant RF2, COG1579 superfamily, ZapB superfamily, SMC prok B superfamily, ATG16 superfamily, HAP1 N superfamily, Smc superfamily, ZapB, bZIP HY5 like, DOG1, bziP superfamily, bZIP HBP1b like, PHA03247 superfamily) (Supplementary File S2, Figure S4).

The structural organization of genes further revealed the arrangement of untranslated regions (UTR) and coding regions (CDS) in 112 *BcbZIP* genes. The gene structure organization showed uneven distribution of UTR and CDS regions, suggesting that the introns and exons were highly variable in *BcbZIP* genes. Subfamily S had 3 genes having both UTR and CDS, while 29 genes had only CDS regions. Subfamily C, G, and A had 14 genes having both UTR and CDS, while 16 genes had only CDS regions. Subfamily I, E, F, H, D, and B had 22 genes having both UTR and CDS, while 27 genes had only CDS regions (Supplementary File S2, Figure S5).

### 2.6. Cis Elements Analysis Indicate the Potential of BcbZIP Genes to Mediate Responses to a Diverse Range of Environmental Stimuli

**Figure 3:**
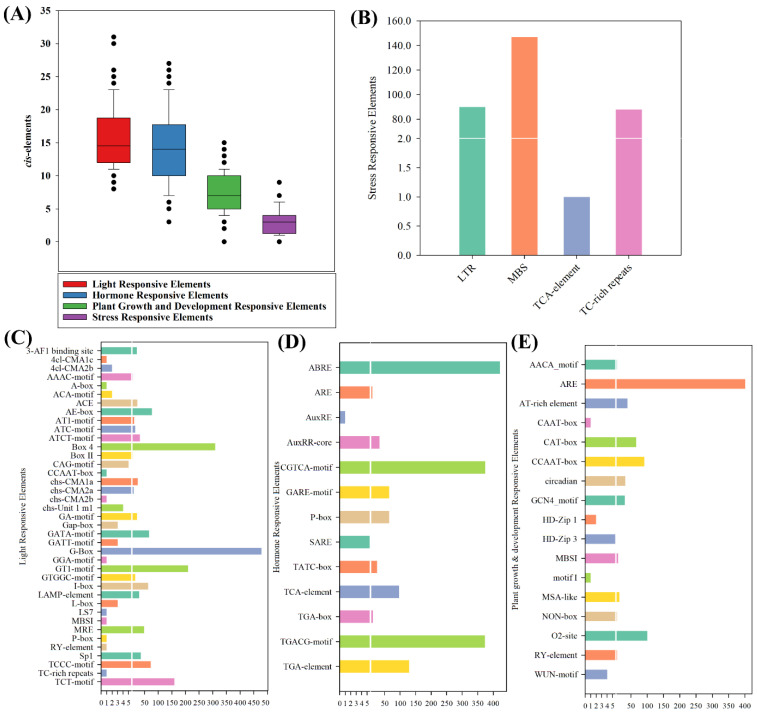
*Cis*-regulatory elements of *BcbZIP* genes. (**A**) Total number of *cis*-elements found in 112 *BcbZIP* genes. (**B**) Stress-responsive elements found in 112 *BcbZIP* genes. (**C**) Light-responsive elements found in 112 *BcbZIP* genes. (**D**) Hormone-responsive elements found in 112 *BcbZIP* genes. (**E**) Plant-growth- and development-responsive elements found in 112 *BcbZIP* genes.

*BcbZIP* genes revealed the presence of numerous *cis*-elements in their 1500 bp upstream promotor sequences, including light, hormone, stress, and plant growth and development related *cis*-elements. Analysis identified a total of 40 photo-responsive *cis*-elements, including 3-AF1 binding site, ACE, GT1 motif. Box-4, I-box, G-box, TCT motif, Sp1, TCCC motif, A-box, AAAC-motif, ACA-motif, AE-box, AT1-motif, ATC-motif, Box II, CAG-motif, CCAAT-box, chs-CMA1a, chs-CMA2a, chs-CMA2b, chs-Unit 1 m1, GA-motif, Gap-box, GATA-motif, GATT-motif, GGA-motif, GTGGC-motif, LAMP-element, L-box, LS7, MBS, MBSI, MRE, P-box, RY-element, TC-rich repeats, 4cl-CMA1c, and 4cl-CMA2b. Additionally, the promoter region contains *cis*-elements that are involved in regulating plant responses to different stresses, including LTR, MBS, TCA-element, and TC-rich repeats. Moreover, 13 hormone-responsive elements like TGA elements. TGA box, ABRE, TATC-box, GARE-motif, ERE, TCA-element, CGTCA-motif, AuxRE, AuxRR-core, ARE, and TGACG-motif, which can bind to regulators in response to hormone signals. Furthermore, 17 *cis*-elements, Circadian, CAT-box, O2-site, MSA-like, AACA motif, ARE, AT-rich element, CAAT-box, GCN4_motif, HD-Zip 1, HD-Zip 3, motif I, WUN-motif, RY-element, CCAAT-box, and MBSI were also identified in the promoter region, which play a crucial role in the binding with regulators that control plant growth and development. The results indicate that *BcbZIP* genes possess the capability to regulate responses to different environmental stimuli. This is attributed to the presence of a wide range of *cis*-elements in their promoter region that are responsive to light, hormones, stress, as well as plant growth and development. (Figure 3).

### 2.7. GO Enrichment Analysis Suggest the Crucial Roles of BcbZIP Genes in Regulating Multiple Cellular Processes

**Figure 4:**
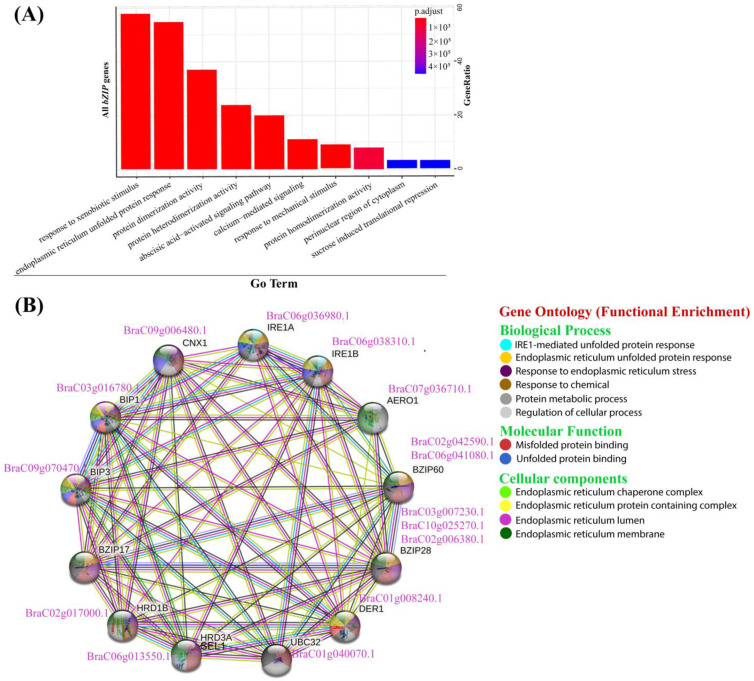
Functional enrichment of *BcbZIP* genes based on gene ontology. (**A**) GO enrichment analysis of 112 *BcbZIP* genes showing their involvement in different biological functions. (**B**) Protein to protein interaction network of *BcbZIP* genes with ER-stress-responsive genes based on their sequence similarities with Arabidopsis. The co-expression network was generated using a string database. The functional annotation of *BcbZIP* genes with ER-stress-responsive genes were represented by different colors of nodes showing the shared biological processes, molecular function, and cellular components.

To further discover the essential role of *BcbZIP* genes in regulating multiple cellular responses, we analyzed the 112 *BcbZIP* genes for GO enrichment. The results of GO enrichment analysis demonstrated that the *BcbZIP* genes share a common set of biological functions. Specifically, a majority of *BcbZIP* genes were identified as being involved in diverse molecular functions and biological processes including, but not limited to, response to xenobiotic stimulus, endoplasmic reticulum-unfolded protein response, protein dimerization, protein heterodimerization, abscisic acid-activated signaling pathway, calcium-mediated signaling, response to mechanical stimulus, and protein homodimerization, as illustrated in (Figure 4A). These findings suggest that the *BcbZIP* genes serve as crucial regulatory factors in a multitude plants cellular processes.

### 2.8. Co-Expression Network Conveyed a Strong Association between BcbZIP Genes with ER Stress Responsive Genes

To further elucidate the contribution of *BcbZIP* genes in ER stress, another BLASTp search was conducted using Arabidopsis protein sequences of ER stress responsive genes (*AT2G17520*, *AT5G24360*, *AT5G28540*, *AT1G09080*, *AT5G61790*, *AT1G72280*, *AT1G65040*, *AT1G18260*, *AT4G29330*, and *AT3G17000* annotated as *ATIRE1A*, *AtIRE1B*, *AtBiP1*, *AtBiP3*, *AtCNX*, *AtERO1 HRD1*, *SEL1*, *DER1*, and *UBC32*, respectively) to identify its homologues in pak choi. Insight into the potential association between *BcbZIP* genes and ER stress responsive genes was obtained through the analysis of *AtbZIP* genes using STRING software v12.0, resulting in a visual map (Figure 4B). The homologues of *BcbZIP* genes based on potential co-expressions of *AtbZIP* genes in the string network confirmed a significant connection between 5 *BcbZIP* genes and 10 ER stress responsive genes, with a *p*-value of <1.0 × 10^−16^. Interaction networks were further analyzed for protein–protein interactions that shared biological processes, molecular function, and cellular components. Edges/lines of varying colors were used to represent strong versus weak interactions among proteins. The colored lines linking the nodes depict the interactions between proteins of the genes, highlighting the protein–protein interactions within the network. These findings revealed a strong association between 5 *BcbZIP* genes and 10 ER-stress-responsive genes.

### 2.9. Expression Profiles of BcbZIP Genes during ER Stress in Pak Choi

**Figure 5:**
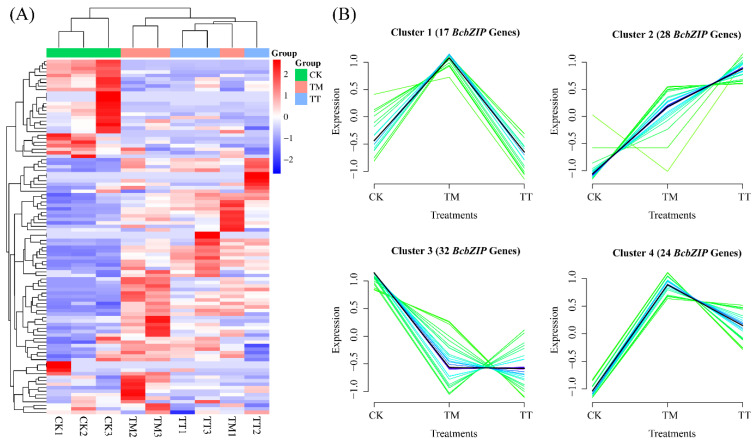
The heat map of TPM expression pattern of *BcbZIP* genes during ER stress (**A**), cluster analysis of all *BcbZIP* genes divided into four clusters based on their expression pattern (**B**). CK: Control, TM: 5 μg/mL, and TT: 25 μg/mL TUDCA application for five days prior to 5 μg/mL TM. Data were recorded after 72 h of TM-induced ER stress.

To explore the expression patterns of the *bZIP* gene family in pak choi under ER stress conditions, we utilized transcriptome profiling data obtained through RNA-Seq analysis. Through this analysis, we observed distinct expression patterns within the *bZIP* genes, which allowed us to classify them into four distinct groups. Notably, genes in groups 1, 2, and 4 exhibited elevated expression levels under ER stress conditions. On the other hand, genes in groups 2 and 3 showed increased expression in the ER stress suppression group, when treated with the ER stress inhibitor, tauroursodeoxycholic acid (TT). These findings provide valuable insights into the dynamic regulation of the *bZIP* gene family in response to ER stress in pak choi (Figure 5 and Supplementary File S3).

### 2.10. Subcellular Localization Revealed the Presence of BcbZIP60a and BcbZIP60b in Nucleus

**Figure 6:**
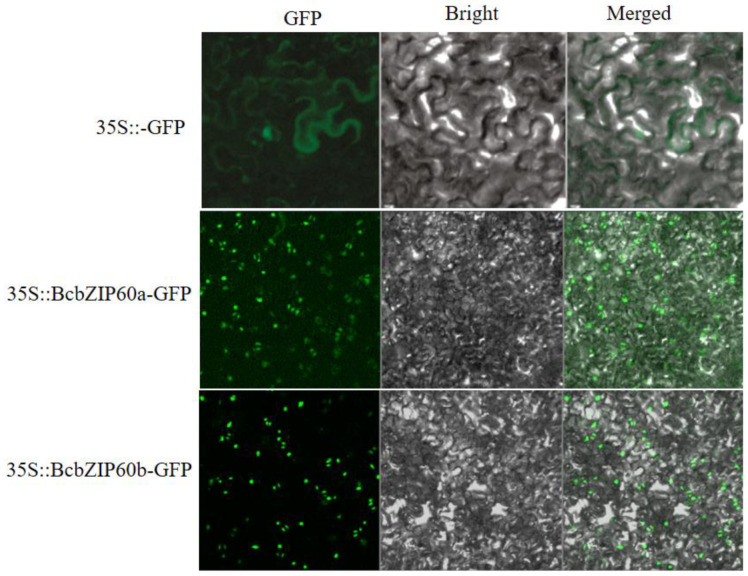
The nuclear localization of *BcbZIP60a* and *BcbZIP60b* investigated in this study.

The subcellular localization of *BcbZIP60a* and *BcbZIP60b* was investigated to gain a better understanding of their functions. In the present study, the leaf cells of *N. benthamiana* were transformed with *BcbZIP60a*: GFP and *BcbZIP60b*: GFP constructs. Confocal laser imaging revealed that both *BcbZIP60a* and *BcbZIP60b*, fused with GFP, emitted green fluorescence in the nucleus of tobacco cells, indicating their localization in the nucleus, suggesting their potential roles in nuclear processes (Figure 6). Understanding the subcellular localization of proteins is crucial for unraveling their functional significance and can contribute to our knowledge of cellular processes and molecular interaction.

### 2.11. Y2H Displayed a Strong Protein–Protein Interaction of BcbZIP60a and BcbZIP60b with BcBiP3

**Figure 7:**
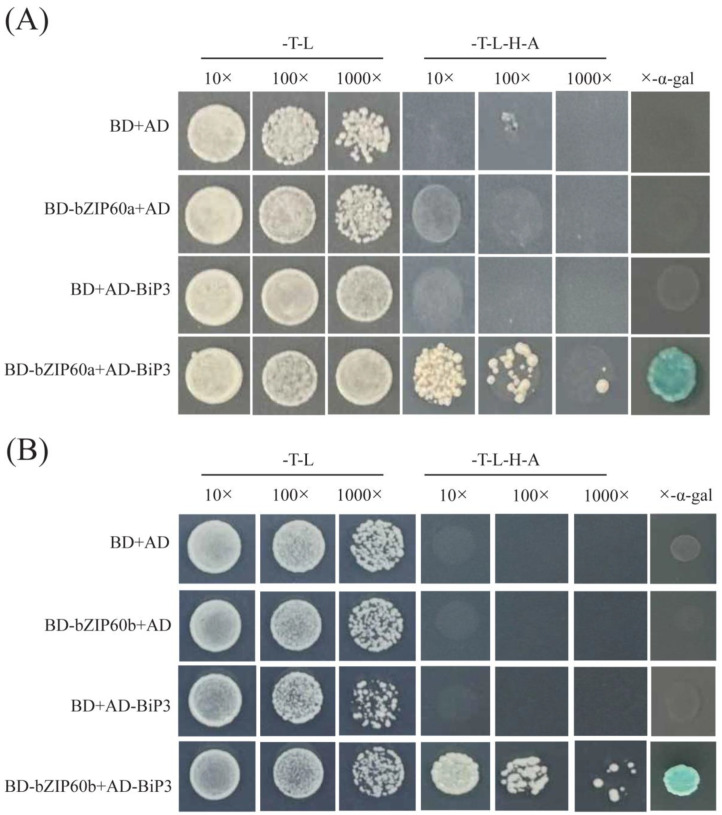
The transcriptional activity of *BcbZIP60a* (**A**) and *BcbZIP60b* (**B**) interacting with *BcBiP* protein, evaluated using X-α-gal assay in yeast (Scale bar = 50 µm), showing positive activity. The positive and negative controls GAL4 and pGBKT7 empty vector were used, respectively.

In this study, the transcription activation of *BcbZIP60a* and *BcbZIP60b* was observed. Recombinant plasmids containing full-length CDS *BcbZIP60a* and *BcbZIP60b* fused with the GAL4 DNA-binding domain (BD-*BcbZIP60a* and BD-*BcbZIP60b*) were constructed and introduced into yeast strains. The results demonstrated that BD-*BcbZIP60a* and BD-*BcbZIP60b* exhibited strong transcription activation activity and activated the expression of reporter genes. This was evident from the growth of yeast colonies on specific medium, while the negative control (BD-PGBKT7 empty vector) showed no growth. Additionally, to investigate the transcriptional mechanism of *BcbZIP60a* and *BcbZIP60b* in response to ER stress, the full-length CDS *BcBiP3* was introduced into the Gal4 activation domain of prey vector (AD-*BcBiP3*). Co-transformation of yeast strains with BD-*BcbZIP60a*, BD-*BcbZIP60b*, and AD-*BcBiP3* revealed that only the yeast co-transformed with *BcbZIP60a* and/or *BcbZIP60b* along with *BcBiP3* could survive under specific conditions. This indicates a strong protein–protein interaction between *BcbZIP60a*, *BcbZIP60b*, and *BcBiP3*. These results highlights valuable insights into the role of *BcbZIP60a* and *BcbZIP60b* as TFs and their involvement in response to ER stress (Figure 7). The study underscores the significance of *bZIP* genes in regulating gene expression and their potential as targets for enhancing plant stress tolerance.

### 2.12. BiFC Assays Indicate the Interaction of BcbZIP60a and BcbZIP60b with BcBiP3 at Multiple Points

**Figure 8:**
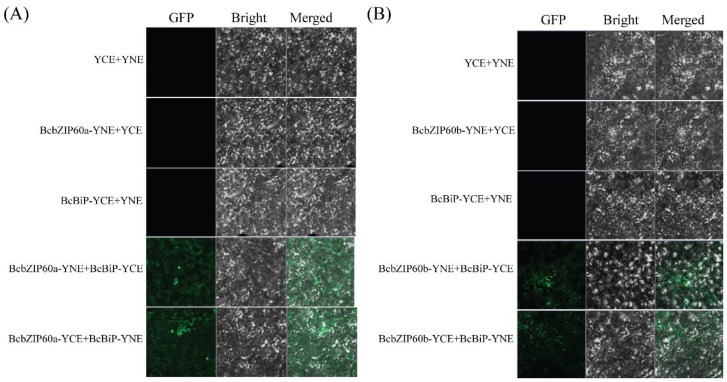
The transcriptional activity of *BcbZIP60a* (**A**) and *BcbZIP60b* (**B**) interacting with *BcBiP* protein in *N. benthamiana* leaf cells on both YNE and YCE terminals.

To investigate the interaction between *BcbZIP60a* and *BcBiP3*, as well as between *BcbZIP60b* and *BcBiP3* in living cells, we employed BiFC assays. The full-length CDS of *BcbZIP60a*, *BcbZIP60b*, and *BcBiP3* were cloned into both YNE and YCE vectors, respectively, to facilitate the BiFC assays. The constructed vectors were then transformed into *A. tumefaciens*. Subsequently, different combinations of these constructs were mixed at 1:1 ratio and infiltrated into one-month-old tobacco epidermal cells. Confocal fluorescence microscopy was used to visualize the results. Positive BiFC interaction signals were observed between *BcbZIP60a*-YNE + *BcBiP*-YCE and *BcbZIP60a*-YCE + *BcBiP*-YNE, indicating an interaction between *BcbZIP60a* and *BcBiP3*. Similarly, GFP signals were detected between *BcbZIP60b*-YNE + *BcBiP*-YCE and *BcbZIP60b*-YCE + *BcBiP*-YNE, suggesting an interaction between *BcbZIP60b* and *BcBiP3*. These findings indicate that both *BcbZIP60a* and *BcbZIP60b* can interact with *BcBiP3* at multiple points, as evidenced by the positive BiFC signals. To serve as negative controls for the BiFC assay, combinations such as YCE + YNE, *BcbZIP60a*-YNE + YCE, *BcBiP*-YCE + YNE, *BcbZIP60b*-YNE + YCE, and *BcBiP*-YCE + YNE were used. None of these combinations exhibited green fluorescence signals in tobacco epidermal cells, further confirming the specificity of the observed interactions (Figure 8).

## 3. Discussion

*bZIP* genes are unique in its structure containing a conserved *bZIP* domain [37]. To date, genome-wide investigations in numerous plants have been used to predict or identify the *bZIP* genes. A total of 75 *bZIP* genes have been identified in *A. thaliana* ([38]. A number of grass family (Gramineae) species have also had their genes characterized, including 187 genes in *Triticum aestivum* [39], 92 in *Sorghum bicolor* [40], 89 in *Oryza sativa* [41], 125 in *Zea mays* [42], and 96 in *Brachypodium distachyon* [43]. Extensive research has led to the discovery and comprehensive examination of numerous *bZIP* genes within brassica species including *B.napus* [44], Chinese cabbage [45], *B. oleraceae* [46], and *B. rapa* [47]. However, despite the extensive research on genomic characterization of *bZIP* genes in multiple plants, our understanding of these genes in pak choi is limited. To address this knowledge gap, we utilized the NHCC database of brassica species to conduct a comprehensive investigation into the genomic characterization of *bZIP* genes in pak choi.

In order to gain a more comprehensive understanding of the evolutionary history of the *BcbZIP* genes in pak choi, the phylogenetic analysis resulted in the clustering of these genes into 10 distinct subfamilies (Figure 2). This classification scheme is consistent with previous findings from similar studies on *bZIP* gene families in numerous other plant species, including Arabidopsis [38], Sorghum [40], Cassava [13], and Grapevine [48]. The most *BcbZIP* genes were found in subgroup S, followed by subgroups A and D, while the least *BcbZIP* genes were found in subgroup H. These findings exhibit similarities to the observations made in *G. biloba* [49], *A. thaliana* [50], Populus [51], *R. sativu* [52], *B. napus*, and *O. europaea* [53], which contains the most *bZIP* members in S, A, I, and D subclasses. This suggests that organization of *bZIP* genes into subfamilies is evolutionarily conserved across different plant species. The classification of *bZIP* genes into subfamilies provides a framework for understanding their functional diversity and potential roles in different biological processes. Moreover, *BcbZIP* genes belonging to the same subgroup exhibit a notable degree of similarity in gene structure, including the number of introns and exons, as well as the distribution of motifs [49].

The organization of exons and introns in a gene indicates the evolutionary path of gene families [54,55]. In this study, an analysis was conducted on the gene structures of 112 *BcbZIP* genes, which revealed a consistent pattern within each group while exhibiting significant variation between different groups (Figure S5). Apart from the *bZIP* domain, several additional domains were also found in *bZIP* genes (Figures S2–S4). These results suggest that various conserved motifs outside the *bZIP* domain could have different roles in determining the functions of bZIP proteins [56,57]. Similarly, *BcbZIP* genes were distributed across all 10 chromosome in pak choi (Figure 3). This distribution is previously been confirmed in *B. rapa* [46] and *B. napus* [44]. Furthermore, tandem/segmental duplications are vital mechanisms that contribute to expansion and diversification of gene families by replicating genes with in the same genomic region or across different chromosomal segments, increasing gene copy numbers. Duplication events are known to play significant roles in gene families expansion [58]. In the current investigation, 38 segmental duplication pairs were recognized in pak choi, as depicted in (Figure 3 and Table 1). These findings suggest that the expansion of the *BcbZIP* gene family primarily originated from segmental duplications. Additionally, it is noteworthy that the Ka/Ks ratios were found to be lower than 1, indicating that these duplicated *BcbZIP* genes likely underwent purifying selection and have been conserved in their functions throughout evolution [59,60].

Functional enrichment analysis is a powerful computational method used to interpret gene expression data and identify pathways and processes associated with specific gene sets or biological entities [61]. According to the results of enrichment analyses of *BcbZIP* genes (Figure 5A). These pathways include hormone responses to xenobiotic stimulus, ER UPR, protein dimerization activity, protein heterodimerization activity ABA-activated signaling pathway, calcium-mediated signaling, mechanical stimulus, and protein homodimerization activity [62,63]. The *BcbZIP* genes have been identified as key regulators in diverse biological processes, including their involvement in the response to ER stress. To gain a deeper understanding of the molecular mechanisms underlying the ER stress response, a protein–protein interaction network analysis was conducted using STRING [59,60,64]. This analysis allowed for the visualization and exploration of protein networks based on functional enrichment analysis (Figure 5B). The ER stress response is primarily intermediated through the UPR pathway, which activates specific genes and signaling pathways.

**Figure 9:**
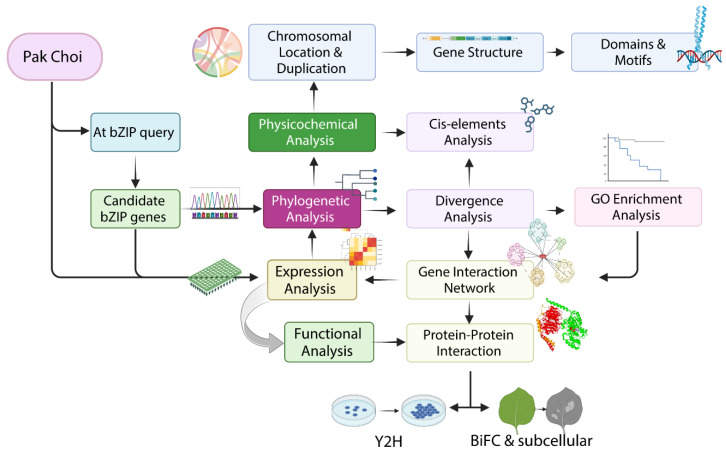
Graphical illustration presenting a comprehensive analysis of molecular characterization of *bZIP* genes, including expression patterns and functional implications during ER stress.

The *bZIP* genes are primarily associated with TFs and play roles in regulating gene expression and important biological processes. These genes are involved in the pathways through which *bZIP* genes control and modulate gene expression [62]. The UPR pathway can be initiated by the accumulation of misfolded proteins within the ER. This pathway involves the participation of *bZIP* genes as well as other genes that are responsive to ER stress (Figure 5B). In the present study, the expression profiles of *BcbZIP* genes were elucidated in response to TM induced ER stress (TM) as well as with the application of TUDCA prior to TM treatment (TT) in pak choi (Figure 6). The expression profiles of most of *BcbZIP* genes were increased in TM. The significant upregulation of these genes in response to TM indicated their involvement in UPR. The protein–protein interaction network of *BcbZIP* genes with ER stress-responsive genes is instrumental in unraveling the physical contacts and interactions among *BcbZIP* and other proteins implicated in ER stress response in the UPR pathway. During ER stress in plant cells, there is an interplay between *bZIP60* and *BiP*. BiP proteins, recognized for their pivotal involvement in protein translocation and folding processes, are prominently induced by the UPR, and serve as a distinctive indicator of ER stress in both animal and plant systems [65,66]. Disruption of protein folding in the ER leads to induction of genes for ER-resident chaperones, including BiP, as part of the ER stress response. However, upon detection of unfolded proteins, BiP is released and binds to unfolded proteins, leaving *IRE1* free to oligomerize and splice *bZIP60* mRNA. This results in the activation of *bZIP60* as a TF [67]. By elucidating these molecular interactions, we further comprehend the intricate regulatory mechanisms at play during ER stress. Correspondingly, to gain a comprehensive understanding of the role of *bZIP* genes in the ER pathway, our study focused on examining the interaction between *BcbZIP60a* and *BcbZIP60b* genes (representatives of *BcbZIP* genes) and the prominent ER chaperone, *BiP3*, which shed light on the molecular mechanisms involved in this pathway. To investigate this interaction, we employed the Y2H assay, which demonstrated a robust association between both *BcbZIP60a* and *BcbZIP60b* with *BcBiP3* (Figure 8). This suggests that *bZIP* genes play an essential role in the regulation of ER function, potentially influencing protein folding, quality control, and ER stress response. To further validate these findings, we conducted a BiFC assay, which provided additional insights into the interaction of both *BcbZIP60a* and *BcbZIP60b* with *BcBiP3*, specifically on both YNE and YCE terminals. This indicates that the interaction between these *bZIP* genes and *BcBiP3* occurs at multiple sites within the ER, further emphasizing their importance in ER homeostasis (Figure 9). *BcbZIP60a* and *BcbZIP60b* are involved in the UPR, which is a signaling pathway that up-regulates the expression of ER chaperones like *BcBiP3* during ER stress. This suggest that under normal conditions, bZIP proteins are bound to the ER membrane and maintained in an inactive state by the chaperone protein BiP. However, during ER stress, *BiP* is sequestered by the accumulation of misfolded proteins, leading to the activation of *bZIP* genes [67]. Once activated, bZIP proteins translocate to the nucleus and bind to specific DNA sequences known as ER stress response elements (ERSEs) present in the promoters of target genes [28]. This binding triggers the up-regulation of genes involved in various aspects of the UPR, including ER-associated degradation (ERAD), which helps in clearing misfolded proteins, and the synthesis of ER chaperones, which assist in protein folding [68]. Our results also confirmed the nuclear localization of *BcbZIP60a* and *BcbZIP60b*, as shown in (Figure 7). Moreover, *bZIP* genes also regulate the expression of genes involved in lipid metabolism, redox homeostasis, and apoptosis [69].

The comprehensive results presented in this research offer compelling evidence of the participation of *BcbZIP* genes in pathways related to ER stress tolerance. Moreover, the functional characterization of these genes highlights their indispensable roles in the UPR. These observations form a crucial foundation for the genetic screening and engineering of novel *bZIP* genes with enhanced ER stress tolerance in pak choi.

## 4. Materials and Methods

### 4.1. Study Layout and Plant Growth Conditions

The Arabidopsis Information Resource (TAIR) database ftp://ftp.arabidopsis.org) (accessed on 12 March 2023), and the newly developed NHCC database http://tbir.njau.edu.cn/NhCCDbHubs/ (accessed on 15 March 2023), were used to retrieve *bZIP* genes from the genomes of *Arabidopsis thaliana* (V.10) and pak choi (V 1.0), respectively. The data obtained were then implemented to undergo *in silico* characterization of *bZIP* genes using advanced bioinformatics analysis. To further perform wet lab experiments for the differential expression levels of *bZIP* genes during ER stress, seeds of pak choi (variety Ziguan) were germinated on pre-soaked filter paper in Petri dishes and subsequently transplanted into vermiculite-containing planting trays in controlled conditions (16 h light and 8 h dark, relative humidity 60%, Av. Temp. 18–24 °C). The plants at 4 to 5 leaf stage were then stratified into three individual groups: those left untreated, served as controls (CK); those induced with ER stress using 5 μg/mL tunicamycin treatment, marked as TM; and plants treated with 25 μg/mL tauroursodeoxycholic acid (TUDCA), as an ER stress suppressor, for 5 days prior to TM treatment, marked as TM + TUDCA [70]. Leaf samples were collected after 72 h of treatment, frozen in liquid nitrogen immediately, and sent to company for RNA sequencing. To conduct a more in-depth analysis of *bZIP* genes role in the ER stress pathway, the chosen *bZIP* genes, along with the highly abundant ER chaperone *BiP3*, were cloned into specific vectors. This was done in order to carry out experiments that investigate protein–protein interactions and determine the subcellular localization of these genes (Figure 9). 

### 4.2. Identification and Physicochemical Analysis of bZIP Genes

Two methods were initiated to find the candidate orthologous of *bZIP* genes in pak choi. Firstly, BLASTp (E-value 1 × 10^−5^) search was conducted using the protein sequences of *AtbZIP* genes as queries keeping the pak choi genome as database to get the candidate orthologous of *bZIP* genes. To further reassure the members of *bZIP* genes in pak choi, a reciprocal BLASTp was conducted using the candidate orthologous of pak choi as queries while keeping the *AtbZIP* genes as candidates. The resulting members were then considered as the homologues of *AtbZIP* genes in pak choi [71]. Secondly, the identified candidate homologues of *AtbZIP* genes in pak choi were further confirmed via HMMER 3.0 http://hmmer.janelia.org/ (accessed on 16 April 2023), using the *bZIP* domain file PF00170.22 against the pak choi genome [64]. To remove redundancy, the candidate homologues were further confirmed by performing bZIP domains annotation via MOTIF search https://www.genome.jp/tools/motif/ (accessed on 16 April 2023), NCBI conserved domain database http://www.ncbi.nlm.nih.gov/Structure/cdd/wrpsb.cgi (accessed on 16 April 2023), SMART database http://smart.embl-heidelberg.de/ (accessed on 16 April 2023), and Pfam database http://pfam.janelia.org/ (accessed on 16 April 2023). The final candidate homologues of *bZIP* genes in pak choi were then annotated as *BcbZIP* genes. Furthermore, the physicochemical properties including amino acids, molecular weight, PI, GRAVY, and formula were determined using the ExPASy-ProtParam tool http://web.expasy.org/protparam/ (accessed on 16 April 2023) [72]. The *in silico* subcellular localization was determined through the use of a predictive tool available at https://predictprotein.org (accessed on 16 April 2023).

### 4.3. Multiple Sequence Alignment and Phylogenetic Analysis of BcbZIP Genes

To further get insights into phylogeny of *BcbZIP* genes, 70 protein sequences of *AtbZIP* genes, except three sequences, i.e., *At3g17610* (*HYH*), *AT2G12980* (*AtbZIP32*), and *AT2G13130* (*AtbZIP73*), which were drawn out because of non-availability, and the 112 of *BcbZIP* protein sequences, except the redundant sequences (*BraC08g022000*.1, *BraC05g001930*.1, *BraC09g063720*.1, *BraC05g009850*.1, *BraC01g002060*.1, *BraC06g034200*.1, *BraC03g018730*.1, and *BraCxxg010020*.1), satisfying all the requirements, were aligned using multiple sequence alignment tool MUSCLE https://www.ebi.ac.uk/Tools/msa/muscle/ (accessed on 2 May 2023) with default settings to get most accurate alignment. The tree was then generated by MEGA 11 software using the neighbor joining model, with 1000 bootstrap values [73].

### 4.4. Chromosomal Location and Duplication of BcbZIP Genes

The chromosomal distribution of 112 *BcbZIP* genes were identified based on the chromosomal information provided in the NHCC database http://tbir.njau.edu.cn/NhCCDbHubs/ (accessed on 17 May 2023). The synteny analysis between 112 *BcbZIP* genes were done using the BLASTp algorithm, and gene duplication or synteny events were evaluated with the Multiple Collinearity Scan toolkit (MCScanX) using a default parameter, and the visualization was created by the circos. The circos plot was then generated via circos visualizing tool in TBtools software v2.056 [74] from the available chromosome length, location of *bZIP* genes on chromosome, and linked genes regions showing the chromosomal location and duplication in pak choi [75].

### 4.5. Gene Structure, Domains, and Motifs

To perform a thorough analysis of the domains present in BcbZIP proteins, the corresponding protein sequences were exposed to domain analysis using the NCBI CDD online software (accessed on 20 May 2023). The resulting predicted domain information was then used for the visualization of domain information using the TBtools software. Additionally, conserved motif analyses of the BcbZIP proteins were carried out using MEME suite software v5.4.1 https://meme-suite.org/meme/tools/meme (accessed on 2 June 2023) [76], identifying 10 conserved motifs, and further visualized via TBtools software. Consequently, to visualize the gene structure organization, TBtools software was used. This involved submitting the gff3 files of the pak choi genome, along with a list of identified gene IDs. This approach provide a comprehensive analysis for the domains, motifs, and gene structure organization of *BcbZIP* proteins. 

### 4.6. Divergence, Cis Elements, and GO Enrichment Analysis

To further check the *BcbZIP* genes for synonymous and non-synonymous changes and their divergence through time, the *Ka*/*Ks* calculator http://services.cbu.uib.no/tools/kaks (accessed on 8 June 2023) [77] was utilized to compute *Ka*/*Ks* values in DNA of *BcbZIP* genes. The divergence time was then calculated in TMY (time in million years) using the following formula;

$$Time\;of\;divergence\;\left(T\right)=\frac{Synonymous\;substitution\;rate\;\left(dS\;\mathrm{or}\;Ks\right)}{2\times Divergence\;rate\;(6.56\times 10^{-9})}\times TMY\;(10^{-6})$$

For specific genes, *cis*-acting regulatory elements were identified by retrieving the promoter sequences of *BcbZIP*, which were 1500 bp upstream, from the pak choi genome based on generic file format (GFF). PlantCARE tool https://bioinformatics.psb.ugent.be/webtools/plantcare/html/ (accessed on 20 June 2023) was used to identify these regulatory elements [78]. The GO enrichment Analysis was done by submitting the gene IDs of *BcbZIP* in NHCC online database http://tbir.njau.edu.cn/NhCCDbHubs/ (accessed on 20 June 2023), using the GO enrichment Analysis tool.

### 4.7. Co-Expression Network Analysis of BcbZIP Genes with ER Stress Responsive Genes

To generate a co-interaction network of *bZIP* genes along with ER stress pathway genes, the protein sequences of the *bZIP* genes and ER stress pathway genes were utilized. This analysis was conducted using String https://cn.string-db.org/ (accessed on 25 June 2023). Furthermore, in order to detect homologous sequences within the pak choi genome, a BLAST search was performed using the protein sequences of key genes implicated in the ER stress pathway in *Arabidopsis* as queries. Homologous sequences were determined based on their substantial e-value of 1 × 10^−10^, and these sequences were subsequently regarded as the most promising hits and selected as homologs. *BcbZIP* genes along with homologues sequences of *Arabidopsis*, were further subjected to co-interaction analysis and functional annotation in String database. This analysis involved leveraging the available transcriptomic data of Arabidopsis to gain insights into the co-expression patterns and potential functional associations of identified genes. The methods employed in this study were based on previously established approaches described by [79].

### 4.8. Expression Analysis of BcbZIP in Response to ER Stress

An experiment was conducted to analyze the expression of *BcbZIP* genes in pak choi under ER stress. The experiment involved three treatment groups: a control group (CK) with no chemical treatment, a group exposed solely to TM to induce ER stress (TM group), and a group that received a TUDCA pretreatment for five days followed by TM application to alleviate ER stress (TM + TUDCA group) [70]. Leaf specimens were collected 72 h after exposure to ER stress, with three independent biological replicates to ensure result reliability and accuracy. The RNA extraction, library construction, and RNA sequencing were outsourced to a service company (Sangon biotech, Shanghai, China). Total RNA was extracted using TRNzol Reagent following the manufacturer’s instructions. The integrity of the extracted RNA was assessed using an Agilent 2100 Bioanalyzer (Agilent Technologies, CA, USA). The nine libraries were sequenced on an Illumina Hiseq platform using 150 bp paired-end sequencing.

### 4.9. Yeast Two Hybrid (Y2H) Assay of BcbZIP60 with ER Chaperone BcBiP3

The Yeast two hybrid (Y2H) assay was performed following the guidelines provided by the Matchmaker Gold. To create prey construct, the CDS of *BcBiP3* was fused with the pGADT7 (AD) vector using the EcoRI and BamHI cleavage sites, resulting in the generation of pGADT7: *BcBiP3*. Similarly, the CDS of *BcbZIP60a* and *BcbZIP60b* were fused with the pGBKT7 (BD) vector to create pGBDT7: *BcbZIP60a* and pGBDT7: *BcbZIP60b* as bait constructs. Following that, the bait and prey plasmids were co-introduced into the Y2H Gold yeast strain and cultured on SD/-Trp Leu medium for 80 h. Subsequently, the cells were transferred to a selective medium, specifically SD/-Trp-His-Leu-Ade, which was supplemented with AbA (400 ng/mL) from Takara (Shiga, Japan). The cells were incubated in this medium for a duration of 80 to 90 h [80]. The primers used for the Y2H assay of *BcbZIP60a*, *BcbZIP60b*, and *BcBiP3* are given in Supplementary File S4.

### 4.10. BiFC Assay of BcbZIP60 with ER Chaperone BcBiP3

The BiFC assay was performed based on a previous study [81]. The coding sequence of *BcbZIP60a*, *BcbZIP60b*, and *BcBiP3* were merged to both the N-terminus of GFP (SPYNE) and the C-terminus of GFP (SPYCE). Subsequently, combinations *BcbZIP60a*-YNE/*BcBiP3*-YCE, *BcbZIP60a*-YCE/*BcBiP3*-YNE, *BcbZIP60b*-YNE/*BcBiP3*-YCE, and *BcbZIP60b*-YCE/*BcBiP3*-YNE were co-injected into tobacco leaves. SPYNE and SPYCE empty vectors were used as negative controls. The emitted green fluorescent protein (GFP) signal was detected using a Confocal Laser Scanning Microscope. The fluorescence was observed at a wavelength of 488 nm. The primers used for the BiFC of *BcbZIP60a*, *BcbZIP60b*, and *BcBiP* are given in Supplementary File S4.

### 4.11. Subcellular Localization of BcbZIP60 

To conduct subcellular localization assays, the coding regions of *BcbZIP60a* and *BcbZIP60b* were inserted into pCAMBIA 1300 vector containing the GFP reporter, resulting in the generation of *BcbZIP60a*-GFP and *BcbZIP60b*-GFP constructs. Additionally, the pCAMBIA 1300 vector was transformed as a control. The recombinant vectors, along with the control vector, were introduced into *A. tumefaciens* strain GV3101 and infiltrated into tobacco leaves. Subsequently, fluorescence signals were observed using a confocal microscope (LSM800, Zeiss, Germany) to determine the subcellular location of the proteins. [80,82]. The primers used for the subcellular localization of *BcbZIP60a* and *BcbZIP60b* are given in Supplementary File S4.

## 5. Conclusions

In conclusion, the phylogenetic analysis discovered that the 112 *BcbZIP* genes were significantly expanded and classified into 10 subfamilies based on sequence similarities. These genes were unevenly distributed across 10 chromosomes and duplicated during evolution, with a divergence analysis confirming their time of divergence ranging from 0.73 to 80.71 MYA. Domain and motif analyses confirmed the presence of *bZIP* domains in *BcbZIP* genes, with gene structural organization revealing highly diverse exon and intron arrangements. Additionally, a wide range of *cis*-elements were detected in the promoter region of *BcbZIP* genes comprising light, hormone, stress, and plant growth and development responsive elements. GO enrichment analysis confirmed their roles in ER UPR. The co-expression network analysis demonstrated a strong association of *BcbZIP* genes with ER-stress-responsive genes. Expression profiles of *BcbZIP* genes and functional identification via protein–protein interaction further supported their involvement in ER stress response in pak choi. Future directions for this study could involve investigating the functional significance of expanded *bZIP* gene families in other plant species and exploring their roles in response to different environmental stimuli. Additionally, studying regulatory mechanisms governing the expression of *bZIP* genes and their interaction networks in greater detail could provide further insights into ER stress and its implications for plant growth and development.

## Figures and Tables

**Table 1:** Non-synonymous (*Ka*) and synonymous (*Ks*) substitution rate and divergence time (MYA) of *BcbZIP* paralogue gene pairs.

S/No.	Paralogue Gene Pairs		*Ka*	*Ks*	*Ka*/*Ks*	Time (MYA)
1	*BraC06g036350.1*	*BraC06g036450.1*	0.002779	0.009631	0.288556	00.73
2	*BraC07g002710.1*	*BraC09g010600.1*	0.042735	0.242491	0.176234	18.48
3	*BraC03g061850.1*	*BraC08g016590.1*	0.073727	0.422454	0.174521	32.20
4	*BraC02g023630.1*	*BraC07g027810.1*	0.132831	0.455651	0.29152	34.73
5	*BraC07g025140.1*	*BraC09g059910.1*	0.069034	0.401945	0.171749	30.64
6	*BraC01g001260.1*	*BraC03g064330.1*	0.153618	0.31279	0.491122	23.84
7	*BraC02g047730.1*	*BraC02g048410.1*	0.021165	0.039781	0.532035	3.03
8	*BraC03g007230.1*	*BraC10g025270.1*	0.090611	0.280135	0.323456	21.35
9	*BraC02g041500.1*	*BraC06g042940.1*	0.052051	0.251405	0.207041	19.16
10	*BraC02g038580.1*	*BraC06g046020.1*	0.074368	0.354414	0.209835	27.01
11	*BraC06g009370.1*	*BraC09g068000.1*	0.072984	0.223345	0.326779	17.02
12	*BraC02g045000.1*	*BraC09g006060.1*	0.101614	0.413633	0.245661	31.53
13	*BraC02g042590.1*	*BraC06g041080.1*	0.069065	0.319646	0.216069	24.36
14	*BraC02g044260.1*	*BraC06g038750.1*	0.089566	0.244669	0.36607	18.65
15	*BraC04g005820.1*	*BraC09g053140.1*	0.298996	0.668932	0.446976	50.99
16	*BraC07g022530.1*	*BraC09g055620.1*	0.077406	0.337156	0.229585	25.70
17	*BraC09g055600.1*	*BraC09g055640.1*	0.001699	0.01187	0.143158	0.90
18	*BraC04g031300.1*	*BraC05g002400.1*	0.110415	0.253654	0.435296	19.33
19	*BraC04g027890.1*	*BraC05g009380.1*	0.044586	0.338605	0.131674	25.81
20	*BraC06g050540.1*	*BraC10g002520.1*	0.308732	1.058908	0.291557	80.71
21	*BraC03g039400.1*	*BraC03g039750.1*	0.017427	0.030102	0.578933	2.29
22	*BraC01g039580.1*	*BraC05g036280.1*	0.046399	0.22363	0.207481	17.04
23	*BraC02g004000.1*	*BraC10g028400.1*	0.045096	0.284274	0.158634	21.67
24	*BraC09g038170.1*	*BraC09g038420.1*	0.01737	0.017292	1.004532	1.32
25	*BraC02g003360.1*	*BraC10g029390.1*	0.050306	0.424763	0.118432	32.38
26	*BraC09g001020.1*	*BraC09g007910.1*	0.172372	0.35456	0.486157	27.02
27	*BraC07g026920.1*	*BraC07g041060.1*	0.09098	0.484017	0.187969	36.89
28	*BraC02g002200.1*	*BraC10g031480.1*	0.119591	0.952579	0.125544	72.61
29	*BraC01g044020.1*	*BraC05g041370.1*	0.024088	0.381676	0.06311	29.09
30	*BraC06g005390.3*	*BraC09g070850.1*	0.04399	0.278365	0.158029	21.22
31	*BraC03g000260.1*	*BraC03g002970.1*	0.015199	0.01931	0.787134	1.47
32	*BraC07g023180.1*	*BraC09g056730.1*	0.055246	0.382918	0.144277	29.19
33	*BraC04g017540.2*	*BraC09g063730.1*	0.213386	0.537255	0.397179	40.95
34	*BraC06g051940.3*	*BraC08g023460.1*	0.110492	0.554996	0.199086	42.30
35	*BraC05g022350.1*	*BraC08g007780.1*	0.063807	0.348792	0.182937	26.58
36	*BraC08g034790.1*	*BraC09g071660.1*	0.064297	0.446193	0.144102	34.01
37	*BraC04g030930.1*	*BraC05g002220.1*	0.042663	0.557869	0.076475	42.52
38	*BraC08g035030.1*	*BraC10g004690.1*	0.069088	0.593214	0.116463	45.21

## Data Availability

The data supporting the results are already given in the main text and Supplementary Files.

## Supplementary Materials

The following supporting information can be downloaded at: https://www.mdpi.com/article/10.3390/plants13081160/s1, The physicochemical properties of 112 identified *BcbZIP* genes (File S1); Domains, motifs, Sequence logos of motifs, and Gene structure Organization of BcbZIP genes (File S2); TPM values of expression of BcbZIP genes (File S3); Primers used in this study (File S4).

## References

[1] Das P., Lakra N., Nutan K.K., Singla-Pareek S.L., Pareek A. (2019). A Unique bZIP Transcription Factor Imparting Multiple Stress Tolerance in Rice. Rice.

[2] Song L., Li W., Chen X. (2022). Transcription Factor Is Not Just a Transcription Factor. Trends Plant Sci..

[3] Pérez-Rodríguez P., Riaño-Pachón D.M., Corrêa L.G.G., Rensing S.A., Kersten B., Mueller-Roeber B. (2010). PlnTFDB: Updated Content and New Features of the Plant Transcription Factor Database. Nucleic Acids Res..

[4] Duan L., Mo Z., Fan Y., Li K., Yang M., Li D., Ke Y., Zhang Q., Wang F., Fan Y. (2022). Genome-Wide Identification and Expression Analysis of the bZIP Transcription Factor Family Genes in Response to Abiotic Stress in *Nicotiana tabacum* L.. BMC Genom..

[5] Hussain S., Tai B., Hussain A., Jahan I., Yang B., Xing F. (2022). Genome-Wide Identification and Expression Analysis of the Basic Leucine Zipper (bZIP) Transcription Factor Gene Family in *Fusarium graminearum*. Genes.

[6] Yoshida T., Fujita Y., Maruyama K., Mogami J., Todaka D., Shinozaki K., Yamaguchi-Shinozaki K. (2015). Four *Arabidopsis* AREB/ABF Transcription Factors Function Predominantly in Gene Expression Downstream of SnRK2 Kinases in Abscisic Acid Signalling in Response to Osmotic Stress. Plant Cell Environ..

[7] Wang Y., Li L., Ye T., Lu Y., Chen X., Wu Y. (2013). The Inhibitory Effect of ABA on Floral Transition Is Mediated by ABI5 in *Arabidopsis*. J. Exp. Bot..

[8] Matiolli C.C., Tomaz J.P., Duarte G.T., Prado F.M., Del Bem L.E.V., Silveira A.B., Gauer L., Corrêa L.G.G., Drumond R.D., Viana A.J.C. (2011). The Arabidopsis bZIP Gene AtbZIP63 Is a Sensitive Integrator of Transient Abscisic Acid and Glucose Signals. Plant Physiol..

[9] Stotz H.U., Mueller S., Zoeller M., Mueller M.J., Berger S. (2013). TGA Transcription Factors and Jasmonate-Independent COI1 Signalling Regulate Specific Plant Responses to Reactive Oxylipins. J. Exp. Bot..

[10] Assunção A.G.L., Herrero E., Lin Y.-F., Huettel B., Talukdar S., Smaczniak C., Immink R.G.H., van Eldik M., Fiers M., Schat H. (2010). *Arabidopsis thaliana* Transcription Factors bZIP19 and bZIP23 Regulate the Adaptation to Zinc Deficiency. Proc. Natl. Acad. Sci. USA.

[11] Dietrich K., Weltmeier F., Ehlert A., Weiste C., Stahl M., Harter K., Dröge-Laser W. (2011). Heterodimers of the *Arabidopsis* Transcription Factors bZIP1 and bZIP53 Reprogram Amino Acid Metabolism during Low Energy Stress. Plant Cell.

[12] Zander M., La Camera S., Lamotte O., Métraux J.-P., Gatz C. (2010). *Arabidopsis thaliana* Class-II TGA Transcription Factors Are Essential Activators of Jasmonic Acid/Ethylene-Induced Defense Responses. Plant J..

[13] Hu W., Yang H., Yan Y., Wei Y., Tie W., Ding Z., Zuo J., Peng M., Li K. (2016). Genome-Wide Characterization and Analysis of bZIP Transcription Factor Gene Family Related to Abiotic Stress in Cassava. Sci. Rep..

[14] Zhou Z., Xiao L., Zhao J., Hu Z., Zhou Y., Liu S., Wu H., Zhou Y. (2023). Comprehensive Genomic Analysis and Expression Profile of *Hsp70* Gene Family Related to Abiotic and Biotic Stress in Cucumber. Horticulturae.

[15] Du K., Huang J., Wang W., Zeng Y., Xuecao L., Zhao F. (2024). Monitoring Low-Temperature Stress in Winter Wheat Using TROPOMI Solar-Induced Chlorophyll Fluorescence. IEEE Trans. Geosci. Remote Sens..

[16] Chambers J.E., Marciniak S.J. (2014). Cellular Mechanisms of Endoplasmic Reticulum Stress Signaling in Health and Disease. 2. Protein Misfolding and ER Stress. Am. J. Physiol.-Cell Physiol..

[17] Bailey D., O’Hare P. (2007). Transmembrane bZIP Transcription Factors in ER Stress Signaling and the Unfolded Protein Response. Antioxid. Redox Signal..

[18] Hetz C., Zhang K., Kaufman R.J. (2020). Mechanisms, Regulation and Functions of the Unfolded Protein Response. Nat. Rev. Mol. Cell Biol..

[19] Liu J.-X., Howell S.H. (2016). Managing the Protein Folding Demands in the Endoplasmic Reticulum of Plants. New Phytol..

[20] Zhang L., Chen H., Brandizzi F., Verchot J., Wang A. (2015). The UPR Branch IRE1-bZIP60 in Plants Plays an Essential Role in Viral Infection and Is Complementary to the Only UPR Pathway in Yeast. PLoS Genet..

[21] Ruberti C., Kim S.-J., Stefano G., Brandizzi F. (2015). Unfolded Protein Response in Plants: One Master, Many Questions. Curr. Opin. Plant Biol..

[22] Wan S., Jiang L. (2016). Endoplasmic Reticulum (ER) Stress and the Unfolded Protein Response (UPR) in Plants. Protoplasma.

[23] Hollien J., Lin J.H., Li H., Stevens N., Walter P., Weissman J.S. (2009). Regulated Ire1-Dependent Decay of Messenger RNAs in Mammalian Cells. J. Cell Biol..

[24] Mishiba K., Nagashima Y., Suzuki E., Hayashi N., Ogata Y., Shimada Y., Koizumi N. (2013). Defects in IRE1 Enhance Cell Death and Fail to Degrade mRNAs Encoding Secretory Pathway Proteins in the *Arabidopsis* Unfolded Protein Response. Proc. Natl. Acad. Sci. USA.

[25] Deng Y., Humbert S., Liu J.-X., Srivastava R., Rothstein S.J., Howell S.H. (2011). Heat Induces the Splicing by IRE1 of a mRNA Encoding a Transcription Factor Involved in the Unfolded Protein Response in Arabidopsis. Proc. Natl. Acad. Sci. USA.

[26] Liu J.-X., Srivastava R., Che P., Howell S.H. (2007). An Endoplasmic Reticulum Stress Response in *Arabidopsis* Is Mediated by Proteolytic Processing and Nuclear Relocation of a Membrane-Associated Transcription Factor, bZIP28. Plant Cell.

[27] Srivastava R., Deng Y., Shah S., Rao A.G., Howell S.H. (2013). BINDING PROTEIN Is a Master Regulator of the Endoplasmic Reticulum Stress Sensor/Transducer bZIP28 in *Arabidopsis*. Plant Cell.

[28] Liu J.-X., Howell S.H. (2010). bZIP28 and NF-Y Transcription Factors Are Activated by ER Stress and Assemble into a Transcriptional Complex to Regulate Stress Response Genes in *Arabidopsis*. Plant Cell.

[29] Howell S.H. (2013). Endoplasmic Reticulum Stress Responses in Plants. Annu. Rev. Plant Biol..

[30] Wang H., Niu H., Zhai Y., Lu M. (2017). Characterization of *BiP* Genes from Pepper (*Capsicum annuum* L.) and the Role of *CaBiP1* in Response to Endoplasmic Reticulum and Multiple Abiotic Stresses. Front. Plant Sci..

[31] Yang X., Srivastava R., Howell S.H., Bassham D.C. (2016). Activation of Autophagy by Unfolded Proteins during Endoplasmic Reticulum Stress. Plant J..

[32] Alvim F.C., Carolino S.M.B., Cascardo J.C.M., Nunes C.C., Martinez C.A., Otoni W.C., Fontes E.P.B. (2001). Enhanced Accumulation of BiP in Transgenic Plants Confers Tolerance to Water Stress. Plant Physiol..

[33] Liebrand T.W.H., Kombrink A., Zhang Z., Sklenar J., Jones A.M.E., Robatzek S., Thomma B.P.H.J., Joosten M.H.A.J. (2014). Chaperones of the Endoplasmic Reticulum Are Required for Ve1-Mediated Resistance to *Verticillium*. Mol. Plant Pathol..

[34] Carvalho H.H., Brustolini O.J.B., Pimenta M.R., Mendes G.C., Gouveia B.C., Silva P.A., Silva J.C.F., Mota C.S., Soares-Ramos J.R.L., Fontes E.P.B. (2014). The Molecular Chaperone Binding Protein BiP Prevents Leaf Dehydration-Induced Cellular Homeostasis Disruption. PLoS ONE.

[35] Li Y., Liu G.-F., Ma L.-M., Liu T.-K., Zhang C.-W., Xiao D., Zheng H.-K., Chen F., Hou X.-L. (2020). A Chromosome-Level Reference Genome of Non-Heading Chinese Cabbage [*Brassica campestris* (syn. *Brassica rapa*) ssp. *chinensis*]. Hortic. Res..

[36] Song X., Li Y., Liu T., Duan W., Huang Z., Wang L., Tan H., Hou X. (2014). Genes Associated with Agronomic Traits in Non-Heading Chinese Cabbage Identified by Expression Profiling. BMC Plant Biol..

[37] Ayaz A., Jalal A., Qian Z., Khan K.A., Liu L., Hu C., Li Y., Hou X. (2024). Investigating the Effects of Tauroursodeoxycholic Acid (TUDCA) in Mitigating Endoplasmic Reticulum Stress and Cellular Responses in Pak Choi. Physiol. Plant..

[38] Jalal A., Ali Q., Manghwar H., Zhu D. (2022). Identification, Phylogeny, Divergence, Structure, and Expression Analysis of A20/AN1 Zinc Finger Domain Containing *Stress-Associated Proteins* (SAPs) Genes in *Jatropha curcas* L.. Genes.

[39] Jalal A., Sun J., Chen Y., Fan C., Liu J., Wang C. (2022). Evolutionary Analysis and Functional Identification of Clock-Associated *PSEUDO-RESPONSE REGULATOR* (*PRRs*) Genes in the Flowering Regulation of Roses. Int. J. Mol. Sci..

[40] Dong Y., Lu J., Liu J., Jalal A., Wang C. (2020). Genome-Wide Identification and Functional Analysis of JmjC Domain-Containing Genes in Flower Development of *Rosa chinensis*. Plant Mol. Biol..

[41] Kumar S., Stecher G., Li M., Knyaz C., Tamura K. (2018). MEGA X: Molecular Evolutionary Genetics Analysis across Computing Platforms. Mol. Biol. Evol..

[42] Chen C., Chen H., Zhang Y., Thomas H.R., Frank M.H., He Y., Xia R. (2020). TBtools: An Integrative Toolkit Developed for Interactive Analyses of Big Biological Data. Mol. Plant.

[43] Liu G., Chai F., Wang Y., Jiang J., Duan W., Wang Y., Wang F., Li S., Wang L. (2018). Genome-Wide Identification and Classification of HSF Family in Grape, and Their Transcriptional Analysis under Heat Acclimation and Heat Stress. Hortic. Plant J..

[44] Bailey T.L., Williams N., Misleh C., Li W.W. (2006). MEME: Discovering and Analyzing DNA and Protein Sequence Motifs. Nucleic Acids Res..

[45] Wang D., Zhang Y., Zhang Z., Zhu J., Yu J. (2010). KaKs_Calculator 2.0: A Toolkit Incorporating Gamma-Series Methods and Sliding Window Strategies. Genom. Proteom. Bioinform..

[46] Lescot M., Déhais P., Thijs G., Marchal K., Moreau Y., Van de Peer Y., Rouzé P., Rombauts S. (2002). PlantCARE, a Database of Plant Cis-Acting Regulatory Elements and a Portal to Tools for in Silico Analysis of Promoter Sequences. Nucleic Acids Res..

[47] Szklarczyk D., Gable A.L., Lyon D., Junge A., Wyder S., Huerta-Cepas J., Simonovic M., Doncheva N.T., Morris J.H., Bork P. (2019). STRING V11: Protein–Protein Association Networks with Increased Coverage, Supporting Functional Discovery in Genome-Wide Experimental Datasets. Nucleic Acids Res..

[48] Liang Y., Xia J., Jiang Y., Bao Y., Chen H., Wang D., Zhang D., Yu J., Cang J. (2022). Genome-Wide Identification and Analysis of *bZIP* Gene Family and Resistance of *TaABI5* (*TabZIP96*) under Freezing Stress in Wheat (*Triticum aestivum*). Int. J. Mol. Sci..

[49] Chen S., Cao H., Huang B., Zheng X., Liang K., Wang G.-L., Sun X. (2022). The WRKY10-VQ8 Module Safely and Effectively Regulates Rice Thermotolerance. Plant Cell Environ..

[50] Zhang L., Ma B., Wang C., Chen X., Ruan Y.-L., Yuan Y., Ma F., Li M. (2022). MdWRKY126 Modulates Malate Accumulation in Apple Fruit by Regulating Cytosolic Malate Dehydrogenase (*MdMDH5*). Plant Physiol..

[51] Yang Z., Sun J., Chen Y., Zhu P., Zhang L., Wu S., Ma D., Cao Q., Li Z., Xu T. (2019). Genome-Wide Identification, Structural and Gene Expression Analysis of the bZIP Transcription Factor Family in Sweet Potato Wild Relative Ipomoea Trifida. BMC Genet..

[52] Li X., Gao S., Tang Y., Li L., Zhang F., Feng B., Fang Z., Ma L., Zhao C. (2015). Genome-Wide Identification and Evolutionary Analyses of bZIP Transcription Factors in Wheat and Its Relatives and Expression Profiles of Anther Development Related *TabZIP* Genes. BMC Genom..

[53] Wang J., Zhou J., Zhang B., Vanitha J., Ramachandran S., Jiang S.-Y. (2011). Genome-Wide Expansion and Expression Divergence of the Basic Leucine Zipper Transcription Factors in Higher Plants with an Emphasis on SorghumF. J. Integr. Plant Biol..

[54] Nijhawan A., Jain M., Tyagi A.K., Khurana J.P. (2008). Genomic Survey and Gene Expression Analysis of the Basic Leucine Zipper Transcription Factor Family in Rice. Plant Physiol..

[55] Wei K., Chen J., Wang Y., Chen Y., Chen S., Lin Y., Pan S., Zhong X., Xie D. (2012). Genome-Wide Analysis of bZIP-Encoding Genes in Maize. DNA Res..

[56] Liu X., Chu Z. (2015). Genome-Wide Evolutionary Characterization and Analysis of bZIP Transcription Factors and Their Expression Profiles in Response to Multiple Abiotic Stresses in Brachypodium Distachyon. BMC Genom..

[57] Zhou Y., Xu D., Jia L., Huang X., Ma G., Wang S., Zhu M., Zhang A., Guan M., Lu K. (2017). Genome-Wide Identification and Structural Analysis of bZIP Transcription Factor Genes in *Brassica napus*. Genes.

[58] Bai Y., Zhu W., Hu X., Sun C., Li Y., Wang D., Wang Q., Pei G., Zhang Y., Guo A. (2016). Genome-Wide Analysis of the bZIP Gene Family Identifies Two ABI5-Like bZIP Transcription Factors, BrABI5a and BrABI5b, as Positive Modulators of ABA Signalling in Chinese Cabbage. PLoS ONE.

[59] Hwang I., Manoharan R.K., Kang J.-G., Chung M.-Y., Kim Y.-W., Nou I.-S. (2016). Genome-Wide Identification and Characterization of bZIP Transcription Factors in Brassica Oleracea under Cold Stress. Biomed. Res. Int..

[60] Hwang I., Jung H.-J., Park J.-I., Yang T.-J., Nou I.-S. (2014). Transcriptome Analysis of Newly Classified bZIP Transcription Factors of Brassica Rapa in Cold Stress Response. Genomics.

[61] Jakoby M., Weisshaar B., Dröge-Laser W., Vicente-Carbajosa J., Tiedemann J., Kroj T., Parcy F. (2002). bZIP Transcription Factors in *Arabidopsis*. Trends Plant Sci..

[62] Liu G.-T., Ma L., Duan W., Wang B.-C., Li J.-H., Xu H.-G., Yan X.-Q., Yan B.-F., Li S.-H., Wang L.-J. (2014). Differential Proteomic Analysis of Grapevine Leaves by iTRAQ Reveals Responses to Heat Stress and Subsequent Recovery. BMC Plant Biol..

[63] Han H., Xu F., Li Y., Yu L., Fu M., Liao Y., Yang X., Zhang W., Ye J. (2021). Genome-Wide Characterization of bZIP Gene Family Identifies Potential Members Involved in Flavonoids Biosynthesis in *Ginkgo biloba* L.. Sci. Rep..

[64] Dröge-Laser W., Snoek B.L., Snel B., Weiste C. (2018). The *Arabidopsis* bZIP Transcription Factor Family-an Update. Curr. Opin. Plant Biol..

[65] Zhao K., Chen S., Yao W., Cheng Z., Zhou B., Jiang T. (2021). Genome-Wide Analysis and Expression Profile of the bZIP Gene Family in Poplar. BMC Plant Biol..

[66] Fan L., Xu L., Wang Y., Tang M., Liu L. (2019). Genome- and Transcriptome-Wide Characterization of *bZIP* Gene Family Identifies Potential Members Involved in Abiotic Stress Response and Anthocyanin Biosynthesis in Radish (*Raphanus sativus* L.). Int. J. Mol. Sci..

[67] Rong S., Wu Z., Cheng Z., Zhang S., Liu H., Huang Q. (2020). Genome-Wide Identification, Evolutionary Patterns, and Expression Analysis of *bZIP* Gene Family in Olive (*Olea europaea* L.). Genes.

[68] Boudet N., Aubourg S., Toffano-Nioche C., Kreis M., Lecharny A. (2001). Evolution of Intron/Exon Structure of DEAD Helicase Family Genes in *Arabidopsis*, *Caenorhabditis*, and *Drosophila*. Genome Res..

[69] Wang N., Zheng Y., Xin H., Fang L., Li S. (2013). Comprehensive Analysis of NAC Domain Transcription Factor Gene Family in Vitis Vinifera. Plant Cell Rep..

[70] Azeem F., Tahir H., Ijaz U., Shaheen T. (2020). A Genome-Wide Comparative Analysis of bZIP Transcription Factors in *G. arboreum* and *G. raimondii* (Diploid Ancestors of Present-Day Cotton). Physiol. Mol. Biol. Plants.

[71] Manzoor M.A., Manzoor M.M., Li G., Abdullah M., Han W., Wenlong H., Shakoor A., Riaz M.W., Rehman S., Cai Y. (2021). Genome-Wide Identification and Characterization of bZIP Transcription Factors and Their Expression Profile under Abiotic Stresses in Chinese Pear (*Pyrus bretschneideri*). BMC Plant Biol..

[72] Wang Q., Guo C., Li Z., Sun J., Wang D., Xu L., Li X., Guo Y. (2021). Identification and Analysis of bZIP Family Genes in Potato and Their Potential Roles in Stress Responses. Front. Plant Sci..

[73] Rathour M., Shumayla, Alok A., Upadhyay S.K. (2022). Investigation of Roles of *TaTALE* Genes during Development and Stress Response in Bread Wheat. Plants.

[74] Sharma H., Sharma A., Rajput R., Sidhu S., Dhillon H., Verma P.C., Pandey A., Upadhyay S.K. (2022). Molecular Characterization, Evolutionary Analysis, and Expression Profiling of *BOR* Genes in Important Cereals. Plants.

[75] Garcia-Moreno A., López-Domínguez R., Villatoro-García J.A., Ramirez-Mena A., Aparicio-Puerta E., Hackenberg M., Pascual-Montano A., Carmona-Saez P. (2022). Functional Enrichment Analysis of Regulatory Elements. Biomedicines.

[76] Ma M., Chen Q., Dong H., Zhang S., Huang X. (2021). Genome-Wide Identification and Expression Analysis of the bZIP Transcription Factors, and Functional Analysis in Response to Drought and Cold Stresses in Pear (*Pyrus breschneideri*). BMC Plant Biol..

[77] Choi J.-W., Kim H.-E., Kim S. (2022). Two Different Domain Architectures Generate Structural and Functional Diversity among bZIP Genes in the Solanaceae Family. Front. Plant Sci..

[78] Bertolotti A., Zhang Y., Hendershot L.M., Harding H.P., Ron D. (2000). Dynamic Interaction of BiP and ER Stress Transducers in the Unfolded-Protein Response. Nat. Cell Biol..

[79] Noh S.-J., Kwon C.S., Oh D.-H., Moon J.S., Chung W.-I. (2003). Expression of an Evolutionarily Distinct Novel BiP Gene during the Unfolded Protein Response in *Arabidopsis thaliana*. Gene.

[80] Srivastava R., Deng Y., Howell S. (2014). Stress Sensing in Plants by an ER Stress Sensor/Transducer, bZIP28. Front. Plant Sci..

[81] Ghemrawi R., Khair M. (2020). Endoplasmic Reticulum Stress and Unfolded Protein Response in Neurodegenerative Diseases. Int. J. Mol. Sci..

[82] Trachootham D., Lu W., Ogasawara M.A., Valle N.R.-D., Huang P. (2008). Redox Regulation of Cell Survival. Antioxid. Redox Signal..

