# Supplementary material for: In-Depth Characterization of bZIP Genes in the Context of Endoplasmic Reticulum (ER) Stress in Brassica campestris ssp. chinensis
Source: Plants (Basel). 2024 Apr 22;13(8):1160. doi: 10.3390/plants13081160 (PMC11053814; doi:10.3390/plants13081160)
Supplement: Supplementary file 1 [file plants-13-01160-s001.zip › Supplementary file S2.pdf]

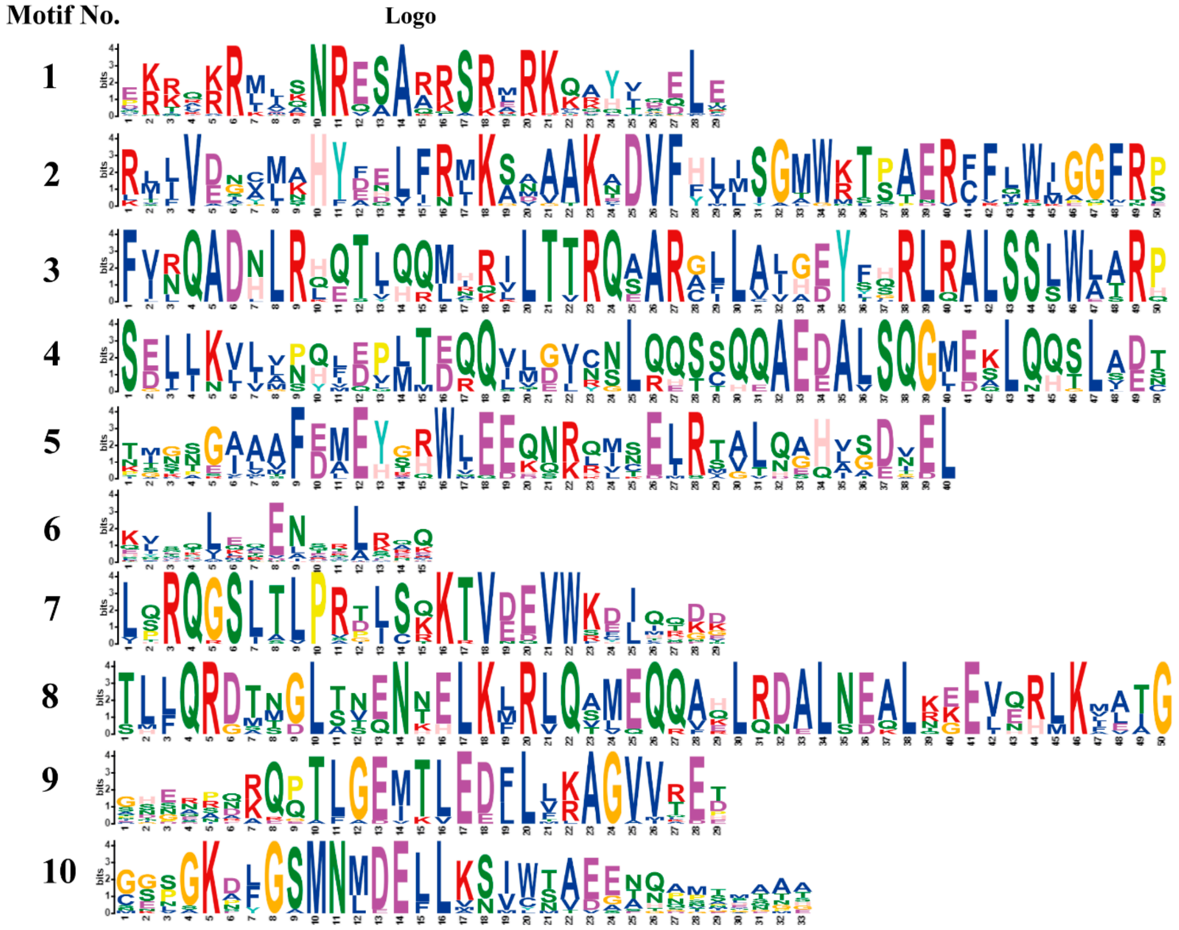

Figure S1. The visual representation of common 10 sequence logos of the conserved motifs in *BcbZIP* genes.

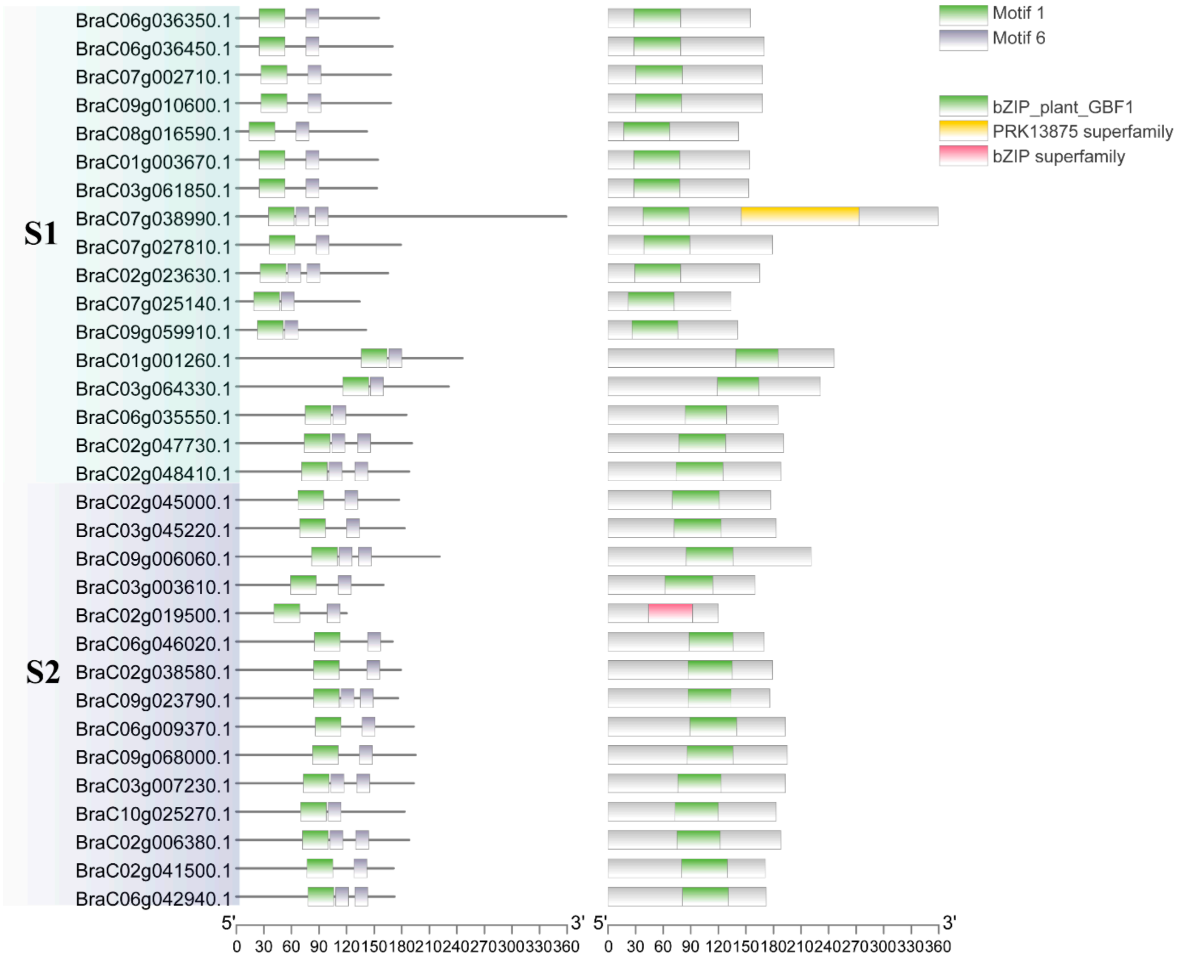

Figure S2. Domain and motif analysis of *BcbZIP* genes of subfamily S (S1 and S2). A representation of the structural characteristics of *BcbZIP* genes, highlighting the presence of *bZIP* domains and specific motifs responsible for their presence.

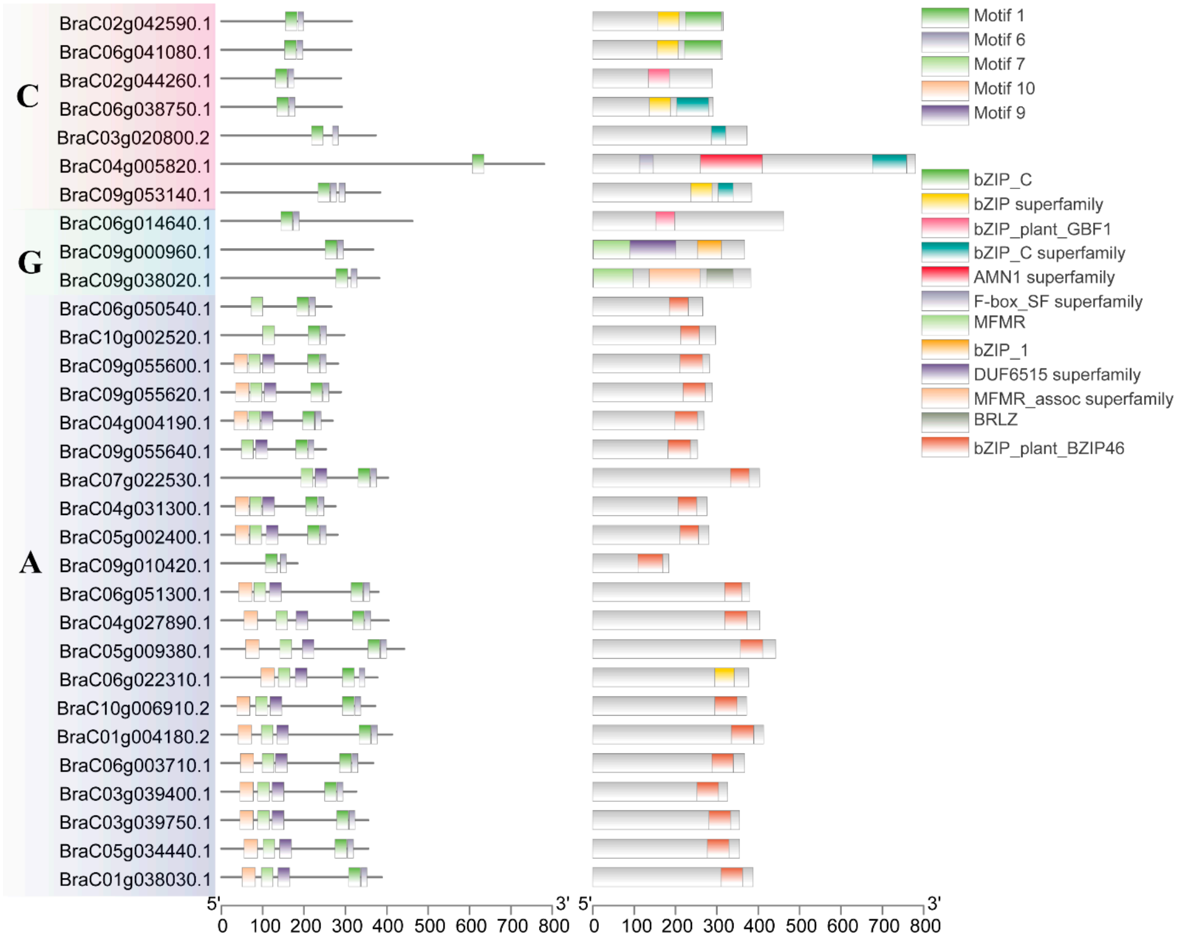

Figure S3. Domain and motif analysis of *BcbZIP* genes of subfamilies C, G, and A. A representation of structural characteristics of *BcbZIP* genes, highlighting presence of *bZIP* domains and specific motifs responsible for their presence.

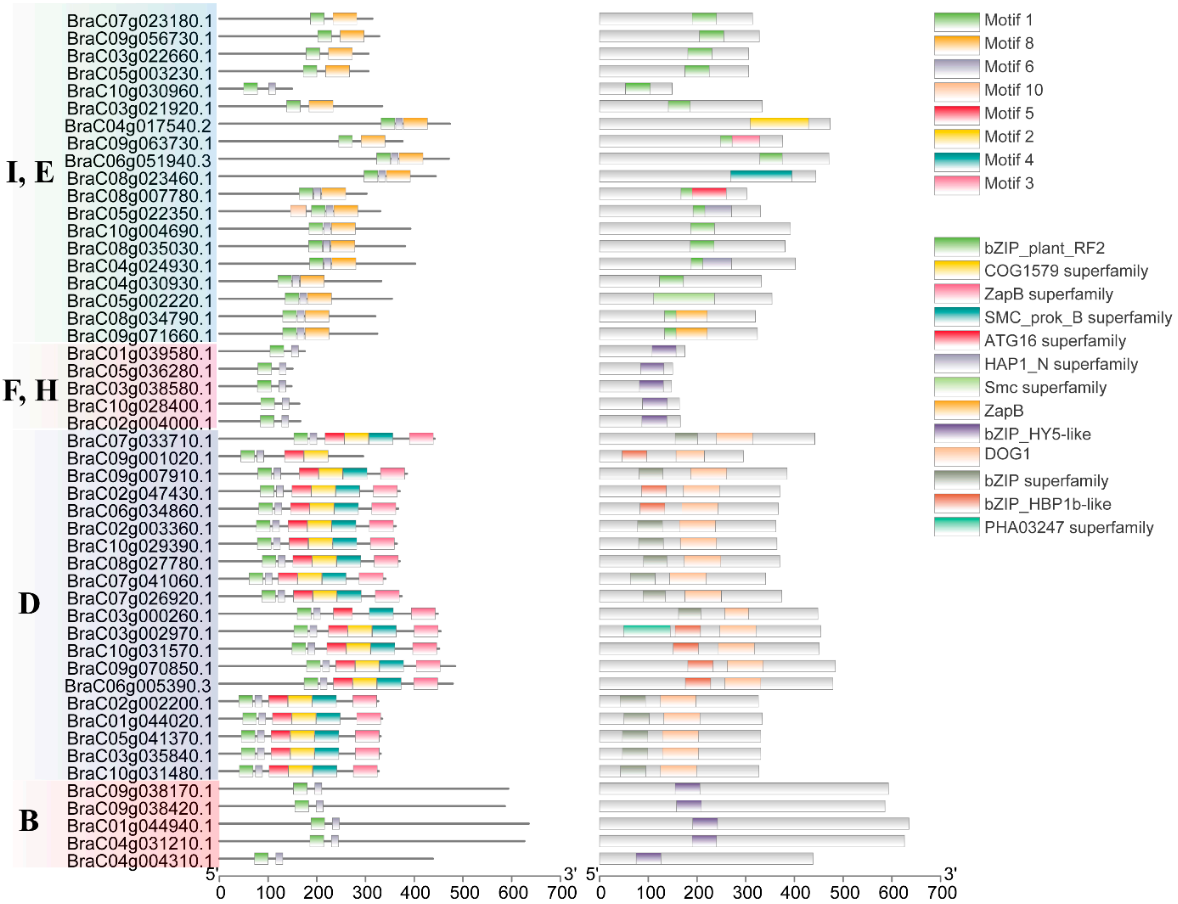

Figure S4. Domain and motif analysis of *BcbZIP* genes of subfamilies I, E, F, H, D, and B. A representation of structural characteristics of *BcbZIP* genes, highlighting presence of *bZIP* domains and specific motifs responsible for their presence.

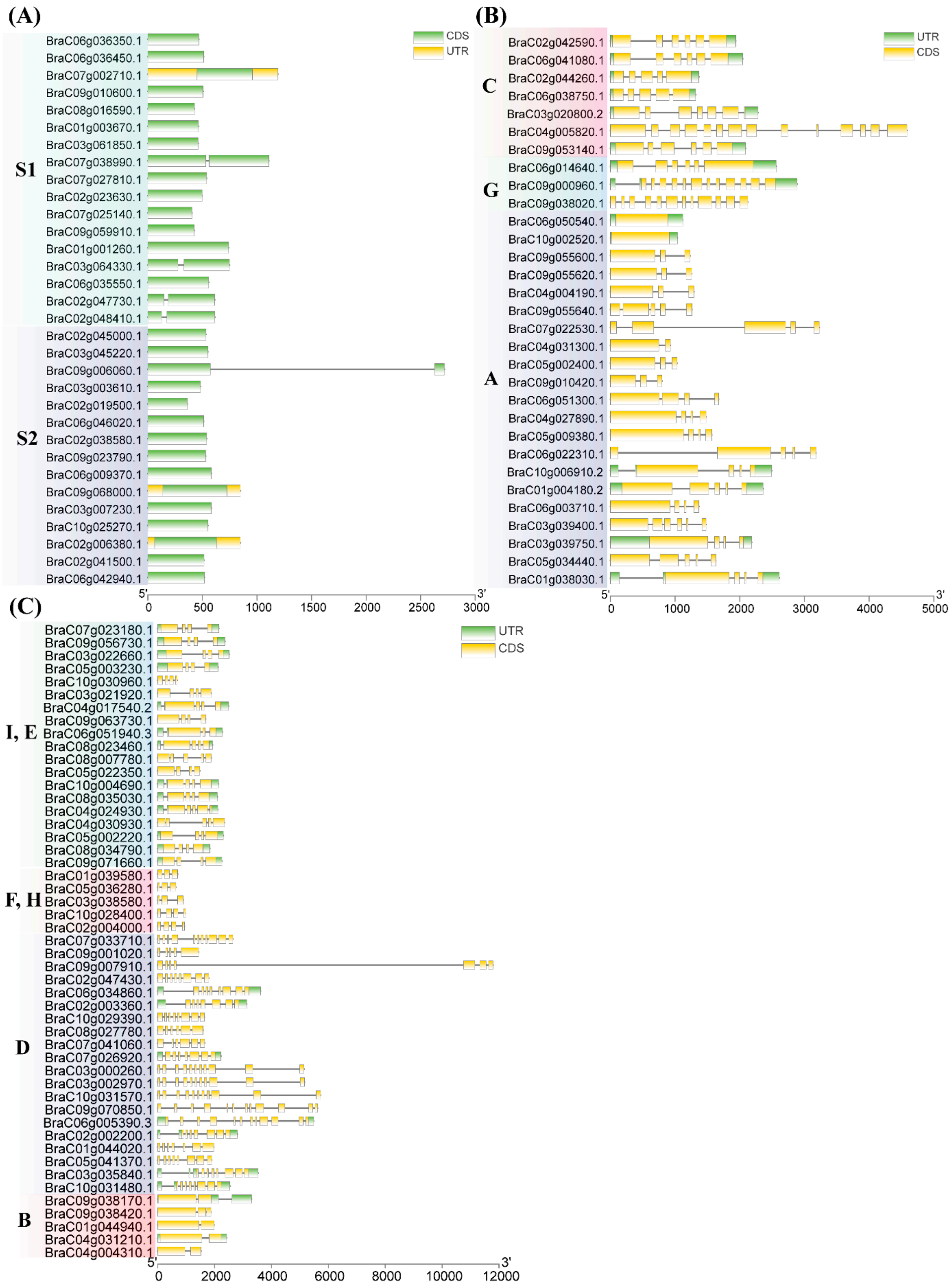

(A)
CDS
UTR
S1
BraC06g036350.1
BraC06g036450.1
BraC07g002710.1
BraC09g010600.1
BraC08g016590.1
BraC01g003670.1
BraC03g061850.1
BraC07g038990.1
BraC07g027810.1
BraC02g023630.1
BraC07g025140.1
BraC09g059910.1
BraC01g001260.1
BraC03g064330.1
BraC06g035550.1
BraC02g047730.1
BraC02g048410.1
S2
BraC02g045000.1
BraC03g045220.1
BraC09g006060.1
BraC03g003610.1
BraC02g019500.1
BraC06g046020.1
BraC02g038580.1
BraC09g023790.1
BraC06g009370.1
BraC09g068000.1
BraC03g007230.1
BraC10g025270.1
BraC02g006380.1
BraC02g041500.1
BraC06g042940.1
5'
0
500
1000
1500
2000
2500
3000
3'
(B)
UTR
CDS
C
BraC02g042590.1
BraC06g041080.1
BraC02g044260.1
BraC06g038750.1
BraC03g020800.2
BraC04g005820.1
BraC09g053140.1
G
BraC06g014640.1
BraC09g000960.1
BraC09g038020.1
A
BraC06g050540.1
BraC10g002520.1
BraC09g055600.1
BraC09g055620.1
BraC04g004190.1
BraC09g055640.1
BraC07g022530.1
BraC04g031300.1
BraC05g002400.1
BraC09g010420.1
BraC06g051300.1
BraC04g027890.1
BraC05g009380.1
BraC06g022310.1
BraC10g006910.2
BraC01g004180.2
BraC06g003710.1
BraC03g039400.1
BraC03g039750.1
BraC05g034440.1
BraC01g038030.1
5'
0
1000
2000
3000
4000
5000
3'
(C)
UTR
CDS
I, E
BraC07g023180.1
BraC09g056730.1
BraC03g022660.1
BraC05g003230.1
BraC10g030960.1
BraC03g021920.1
BraC04g017540.2
BraC09g063730.1
BraC06g051940.3
BraC08g023460.1
BraC08g007780.1
BraC05g022350.1
BraC10g004690.1
BraC08g035030.1
BraC04g024930.1
BraC04g030930.1
BraC05g002220.1
BraC08g034790.1
BraC09g071660.1
F, H
BraC01g039580.1
BraC05g036280.1
BraC03g038580.1
BraC10g028400.1
BraC02g004000.1
D
BraC07g033710.1
BraC09g001020.1
BraC09g007910.1
BraC02g047430.1
BraC06g034860.1
BraC02g003360.1
BraC10g029390.1
BraC08g027780.1
BraC07g041060.1
BraC07g026920.1
BraC03g000260.1
BraC03g002970.1
BraC10g031570.1
BraC09g070850.1
BraC06g005390.3
BraC02g002200.1
BraC01g044020.1
BraC05g041370.1
BraC03g035840.1
BraC10g031480.1
B
BraC09g038170.1
BraC09g038420.1
BraC01g044940.1
BraC04g031210.1
BraC04g004310.1
5'
0
2000
4000
6000
8000
10000
12000
3'

Figure S5. Gene Structure Organization of *BcbZIP* genes. Here; CDS is the coding region, UTR is the untranslated region. (A) Gene structure organization of subfamily S (S1 and S2). (B) Gene structure organization of subfamily C, G, and A. (C) Gene structure organization of subfamily I, E, F, H, D, and B.

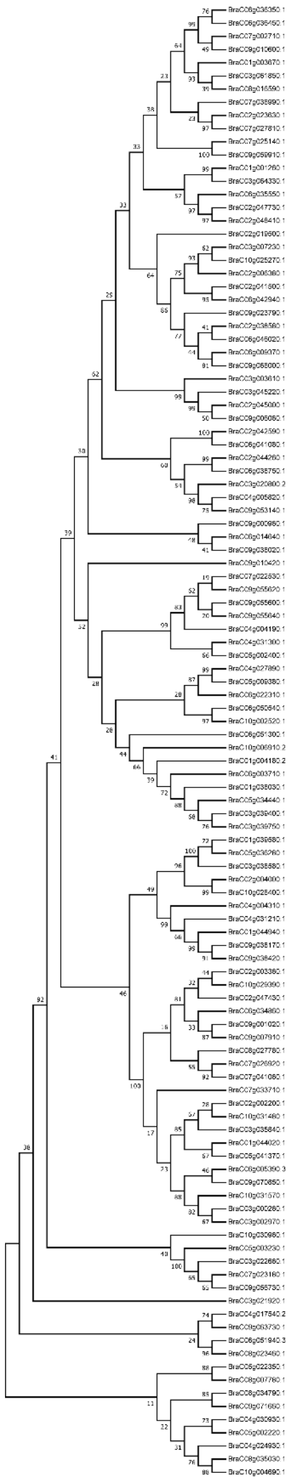

Figure S6: The 38 BcbZIP paralogous genes identified via Ka/Ks calculator.
